# *In situ* generation of photoactivatable aggregation-induced emission probes for organelle-specific imaging†
†Electronic supplementary information (ESI) available: Synthetic procedures, NMR spectra, photophysical characterization data, UV-Vis and PL spectra, cell imaging, and cell viability (PDF). See DOI: 10.1039/c8sc01887a


**DOI:** 10.1039/c8sc01887a

**Published:** 2018-06-01

**Authors:** Shiwu Li, Xia Ling, Yuhan Lin, Anjun Qin, Meng Gao, Ben Zhong Tang

**Affiliations:** a Guangdong Innovative Research Team , Center for Aggregation-Induced Emission , State Key Laboratory of Luminescent Materials & Devices , South China University of Technology , Guangzhou 510640 , China; b School of Medicine , South China University of Technology , Guangzhou 510006 , China; c National Engineering Research Center for Tissue Restoration and Reconstruction , South China University of Technology , Guangzhou 510006 , China . Email: msmgao@scut.edu.cn; d Department of Chemistry , Hong Kong Branch of Chinese National Engineering Research Center for Tissue Restoration and Reconstruction , The Hong Kong University of Science & Technology , Clear Water Bay , Kowloon , Hong Kong , China . Email: tangbenz@ust.hk

## Abstract

Photoactivatable fluorescent probes are ideal tools for organelle study with a significant advantage of high spatiotemporal resolution. However, conventional photo-caged fluorophores for organelle-specific imaging suffer from several drawbacks, such as aggregation-caused quenching (ACQ), instability under ambient light, low photoactivation efficiency, and toxic photo-cleavage byproducts. Herein, we propose a strategy for *in situ* generation of photoactivatable aggregation-induced emission (AIE) probes of 2-(2-hydroxyphenyl)-benzothiazolines from easily available disulfide and thiol substrates through tandem S–S bond reduction and intramolecular cyclization reaction. Because the photoactivatable AIE probes can be *in situ* generated in a quantitative yield, they can be directly used for bio-imaging without complicated separation steps. Under both one- and NIR two-photon irradiation, excellent spatiotemporal resolution and high photoactivation efficiency were achieved for specific imaging of lipid droplets and lysosomes, respectively. Based on their *in situ* generation and adjustable organelle-targeting ability, the photoactivatable AIE probes could become an easy-to-use imaging tool in the study of the biological functions of organelles.

## Abstract 2

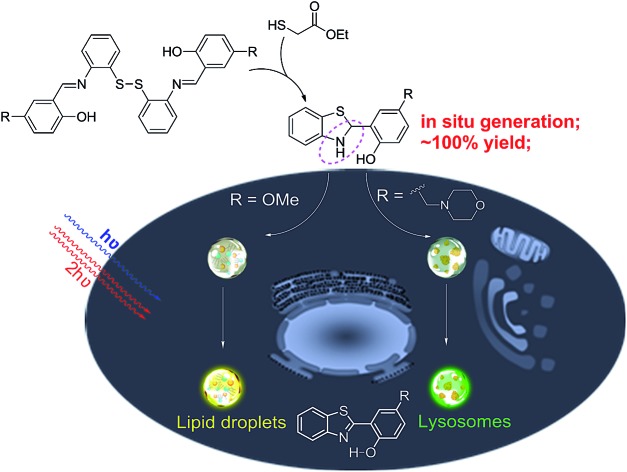
Photoactivatable fluorescent probes are ideal tools for organelle study with a significant advantage of high spatiotemporal resolution.

## Introduction

The intracellular locations and motilities of organelles are closely related to their biological functions.^1^ It's thus highly desirable to track organelle distribution and movement within living cells to reveal their functions in different biological processes.^2^ To date, many “always-on” fluorescent probes have been reported for organelle-specific imaging, but the lack of spatiotemporal controlling ability has seriously restricted their applications.^3^ In contrast, photoactivatable fluorescent probes can transform from a non-emissive state to a highly emissive state under light irradiation with high spatiotemporal resolution, making them powerful imaging tools for organelle study.^4^ In the past few years, a series of caged proteins and fluorophores have been developed for photoactivatable imaging;^5^ however, they suffer from several drawbacks in terms of difficult synthesis, low photoactivation efficiency, inadvertent activation under ambient light, and generation of photocleavage toxic byproducts.^6^ Moreover, conventional fluorophores can easily undergo serious self-quenching after accumulation in organelles, which further restricts their application in organelle study.^7^

Recently, aggregation-induced emission (AIE) has been proposed as a fundamental solution to tackle the challenge of fluorescence self-quenching.^8^ Based on their unique advantages of high brightness in the aggregate state, strong photostability, and low cytotoxicity,^9^ AIE-active probes have found broad applications in bio-imaging and sensing.^10^ Recently, several photoactivatable or photoswitchable AIE fluorogens (AIEgens) have been developed, including tetraphenylethene, cyanostilbene, dihydro-2-azafluorenone, and distyrylanthracene derivatives.^11^ However, these AIEgens need several synthetic steps for preparation and tedious column chromatography procedures for separation. To make photoactivatable probes easy-to-use for organelle study, the following requirements need be satisfied: (1) the photoactivatable probes should be *in situ* generated in a quantitative yield from easily available substrates and directly used for bio-imaging without complicated separation steps; (2) the targeting groups for different organelles should be easily introduced; (3) the photoactivation process should be conducted under NIR two-photon irradiation to reduce the photodamage effect and guarantee high spatiotemporal resolution; and (4) the fluorescence self-quenching effect for organelle imaging should be overcome.

**Scheme 1:**
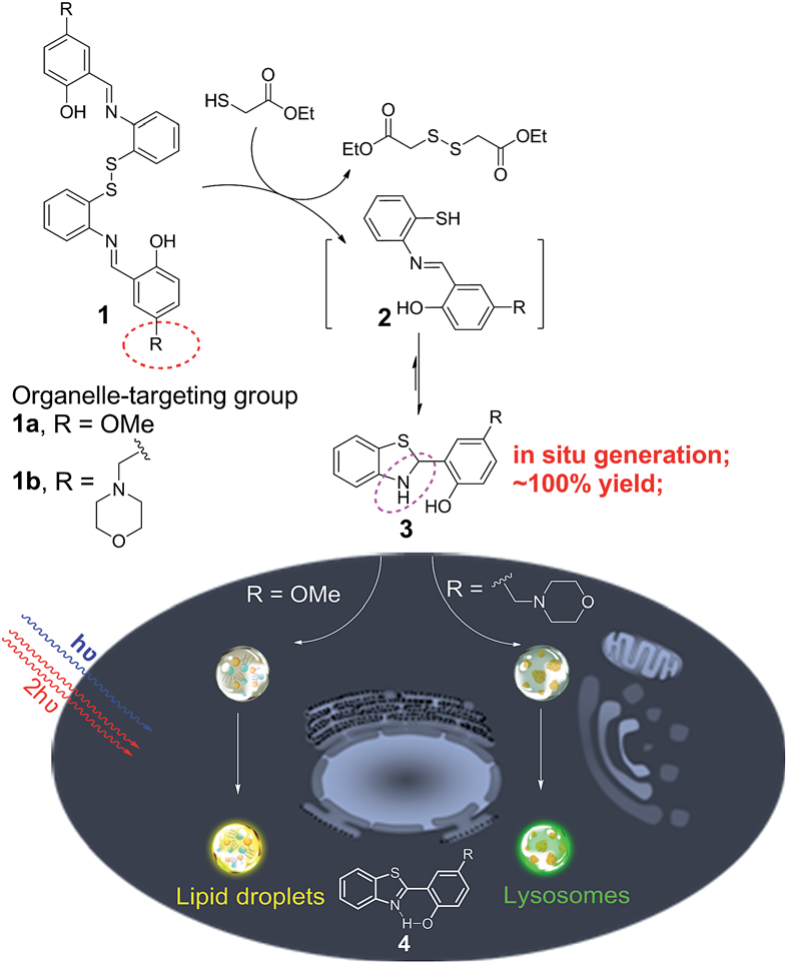
The *in situ* generation of photoactivatable AIE probe **3** for specific imaging of lipid droplets and lysosomes.

Herein, we unexpectedly found that bis(2-(2-hydroxybenzylidene)amino)aryl disulfides **1** and ethyl mercaptoacetate can easily undergo S–S bond reduction to afford Schiff base intermediate **2**, which then underwent intramolecular cyclization reaction to *in situ* quantitatively generate 2-(2-hydroxyphenyl)-benzothiazolines **3**. Compounds **3** can further undergo photooxidative dehydrogenation reaction to afford the AIE-active compound 2-(2-hydroxyphenyl)-benzothiazoles **4** under both one- and two-photon irradiation (Scheme 1). The *in situ* generated compounds **3** with a methoxy or morpholinomethyl substituent can be respectively used for photoactivatable imaging of lipid droplets (LDs) and lysosomes with high specificity and excellent photoactivation efficiency. Because the disulfide and thiol substrates are easily available and the photoactivatable probes **3** could be *in situ* generated in a quantitative yield, they would be easy-to-use tools for organelle study.

## Experimental section

### The *in situ* preparation of photoactivatable AIE probe **3**

Disulfide **1** and ethyl mercaptoacetate were first dissolved in DMSO solutions at a concentration of 10 mM and 20 mM, respectively. Their DMSO solutions were then mixed with an equal volume (100 μL) to *in situ* generate the photoactivatable probe **3** (10 mM, 200 μL) in a quantitative yield within 5 min.

### Cell culture

HeLa and MCF-7 cells were cultured in DMEM containing 1% penicillin–streptomycin and 10% FBS at 37 °C in a humid atmosphere with 5% CO_2_.

### Photoactivatable imaging of lipid droplets

The HeLa or MCF-7 cells were first treated with oleic acid (50 μM) for 6 h to induce the formation of lipid droplets. The cell culture medium was removed and the cells were washed twice with HBSS buffer. Then the *in situ* generated compound **3a** in DMSO solution (5.0 μL, 10 mM) was directly added into the HBSS buffer (1.0 mL) and incubated with the cells for 5 min. Fluorescence images were then taken under a confocal microscope through continuous irradiation at 405 nm with 10% laser power, *λ*_em_ = 500–700 nm.

### Photoactivatable imaging of lysosomes

The *in situ* generated compound **3b** in DMSO solution (2.0 μL, 10 mM) was directly added into the HBSS buffer (1.0 mL) and incubated with the cells for 5 min. Fluorescence images were then taken under a confocal microscope through continuous irradiation at 405 nm with 5% laser power, *λ*_em_ = 450–657 nm.

### Colocalization experiment

For co-staining of compound **3a** and BODIPY493/503 Green, the HeLa or MCF-7 cells in HBSS buffer were first incubated with BODIPY493/503 Green (1.0 μg mL^–1^) for 10 min and then incubated with **3a** (50 μM) for 5 min. The fluorescence images of **3a** after photoactivation were taken with *λ*_ex_ = 405 nm and *λ*_em_ = 550–700 nm. For BODIPY493/503 Green, *λ*_ex_ = 488 nm and *λ*_em_ = 500–550 nm.

For co-staining of compound **3b** and LysoTracker Red, the HeLa or MCF-7 cells in HBSS buffer were first incubated with **3b** (20 μM) for 5 min and then incubated with LysoTracker Red (100 nM) for 5 min. The fluorescence images of **3b** after photoactivation were taken with *λ*_ex_ = 405 nm and *λ*_em_ = 410–539 nm. For LysoTracker Red, *λ*_ex_ = 543 nm and *λ*_em_ = 576–657 nm.

### Photoactivatable imaging under two-photon irradiation

The HeLa or MCF-7 cells stained with the *in situ* generated **3** were irradiated in a bleach model with a two-photon femtosecond laser at 780 nm (1.0% power). The emission filter was 575–630 nm and 495–540 nm for **3a** and **3b**, respectively.

### Spatiotemporal imaging with two-photon irradiation

The fluorescence image of multi-cells was first taken under two-photon irradiation (780 nm, 0.5% laser power). The selected cells were then continuously irradiated with a bleaching model (780 nm, 1.0% laser power) to activate fluorescence. Then, the whole observation window of multi-cells was imaged. This process was repeated to light-up all the selected cells. The emission filter was 575–630 nm and 495–540 nm for **3a** and **3b**, respectively.

## Results and discussion

Compounds **1**, **3**, and **4** can be easily prepared by condensation or cyclization reaction between 2-hydroxybenzaldehyde derivatives and 2,2′-disulfanediyldianiline or 2-aminobenzenethiol (for detailed synthetic procedures, see Scheme S1†).^12^ The structures of all these compounds were unambiguously verified by NMR and HRMS analysis (Fig. S1–S6†).

The AIE properties of compounds **4** were first investigated. For 4-methoxy-substituted compound **4a**, a weak “enol” emission at 404 nm was observed in THF solution, while a strong “keto” emission at 570 nm was observed in the solid state (Fig. S7†). Compared with the THF solution, the fluorescence quantum yield and lifetime of **4a** showed a 33.4-fold increase from 1.2 to 40.1% and a 5.2-fold increase from 0.94 to 4.88 ns, respectively (Table S1†). For 4-morpholinomethyl-substituted compound **4b**, a weak “enol” emission at 446 nm in THF solution and a strong “keto” emission at 523 nm in the solid state were observed. The fluorescence quantum yield and lifetime of **4b** respectively showed a 25.8-fold increase from 1.1% to 28.4% and a 4.1-fold increase from 1.56 to 6.39 ns (Table S1†). The AIE properties of compounds **4** can be ascribed to the synergistic effects of restriction of intramolecular motion (RIM) and excited state intramolecular proton transfer (ESIPT) mechanisms.^13^ The excellent “keto” emission efficiency of compounds **4** in the aggregate state with large Stokes shifts (up to 208 nm) would greatly favor their bio-imaging applications.

**Fig. 1:**
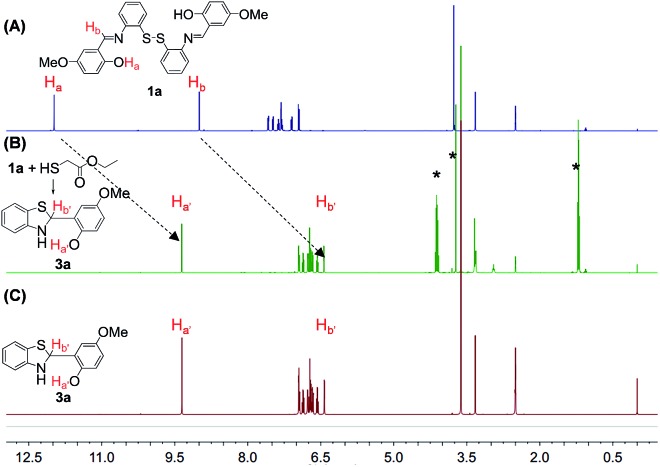
The ^1^H NMR stacking spectra of (A) **1a**, (B) “**1a** + ethyl thioglycolate”, and (C) purified **3a** (* represents diethyl 2,2′-disulfanediyldiacetate).

We then investigated the *in situ* generation ability of compounds **3**. To our satisfaction, a quantitative transformation to **3** was achieved by a simple mixing of the DMSO solutions of disulfides **1** and ethyl mercaptoacetate within 5 min. This quantitative transformation was clearly verified by the NMR analysis of the reaction mixtures. As shown in Fig. 1, the O–H_a_ and C–H_b_ peaks of **1a** at 12.0 and 9.0 ppm completely disappeared after reaction with ethyl mercaptoacetate, and new O–H_a′_ and C–H_b′_ peaks appeared at 9.4 and 6.4 ppm, which overlap well with the purified compound **3a**. Meanwhile, the ethyl mercaptoacetate was oxidized to diethyl 2,2′-disulfanediyldiacetate (Fig. S8†). The *in situ* generation of compound **3a** was also verified by UV-Vis absorption measurements. The main absorption peak of **1a** at 380 nm disappeared after reaction with ethyl mercaptoacetate, and a new absorption peak at 309 nm was observed, which overlaps well with the absorption spectrum of purified compound **3a** (Fig. S9†). Similarly, **3b** could also be *in situ* generated in a quantitative yield by the simple mixing of **1b** and ethyl mercaptoacetate in DMSO solution (Fig. S10†). The quantitative transformation to compound **3** was also verified by the HPLC experiment (Fig. S11†).

**Fig. 2:**
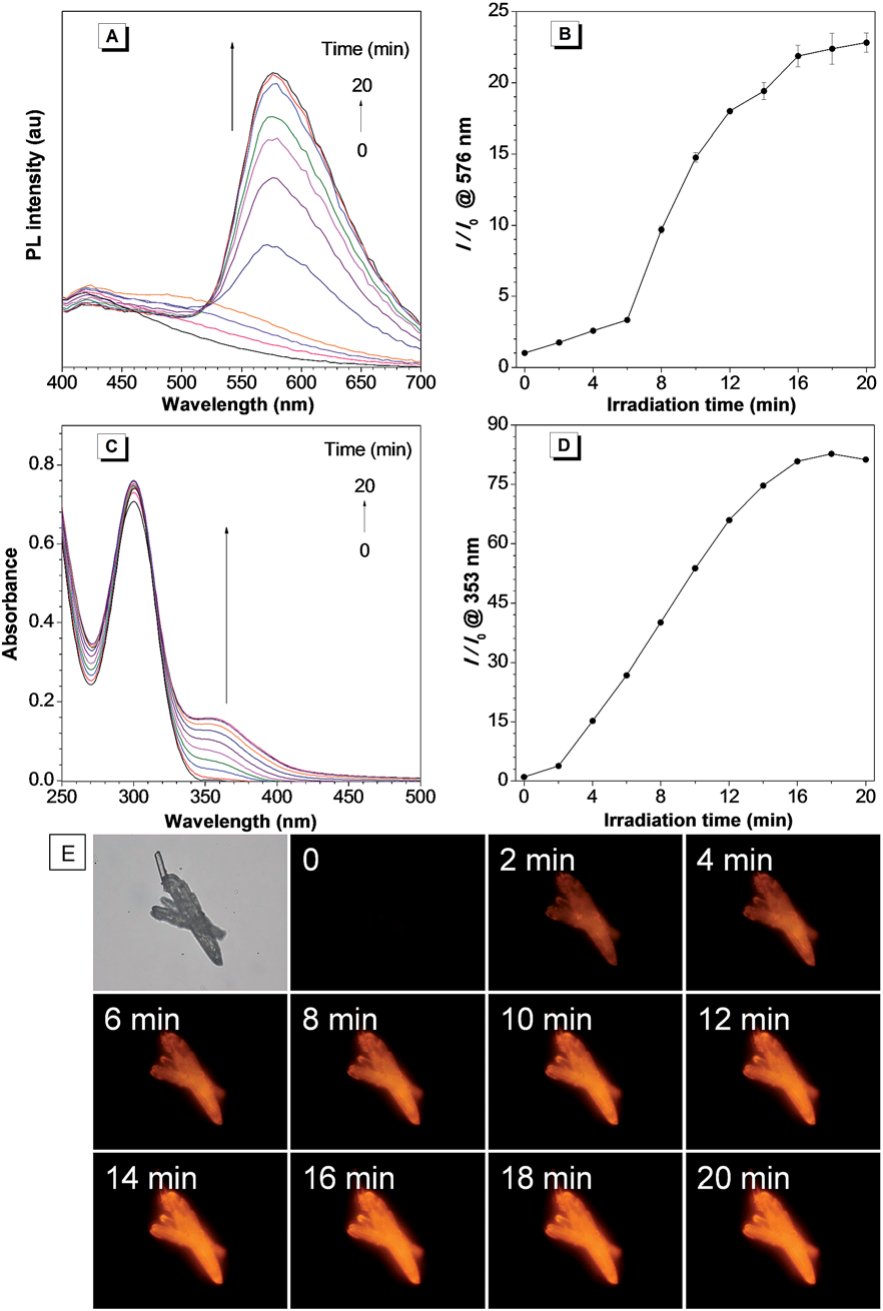
(A) The PL spectra of *in situ* generated **3a** in aqueous solution under irradiation at 365 nm for 0–20 min. (B) Plot of relative PL intensity *I*/*I*_0_ at 576 nm *versus* the irradiation time. (C) The UV-Vis spectra of *in situ* generated **3a** in aqueous solution under irradiation at 365 nm for 0–20 min. (D) Plot of the relative UV-Vis absorption intensity *I*/*I*_0_ at 353 nm *versus* the irradiation time. [**3a**] = 100 μM. (E) Bright-field and fluorescence images of **3a** in the solid state taken under white light and UV irradiation (365 nm).

The photoactivation ability of the *in situ* generated compound **3** was then investigated. Under UV irradiation at 365 nm, the PL intensity of the *in situ* generated **3a** increased quickly with a maximum emission wavelength at 576 nm (Fig. 2A and B). Meanwhile, the absorption intensity of **3a** at 353 nm also increased rapidly (Fig. 2C and D). The PL and UV-Vis spectral changes can be attributed to the generation of compound **4a** through photooxidative dehydrogenation of the C–N single bond to a C=N double bond. The ^1^H NMR spectral analysis further verified the photooxidative dehydrogenation transformation by disappearance of the hydrogen peaks of the C–N single bond (Fig. S12†). Moreover, a gradual light-up fluorescence was observed for compound **3a** in the solid state under UV irradiation (365 nm), which suggests that the photooxidative dehydrogenation reaction can also smoothly proceed in the aggregate or solid state (Fig. 2E). Compound **3b** can also smoothly undergo photooxidative dehydrogenation reaction to efficiently afford the AIE-active compound **4b** both in solution and in the solid state (Fig. S13–S15†).

**Fig. 3:**
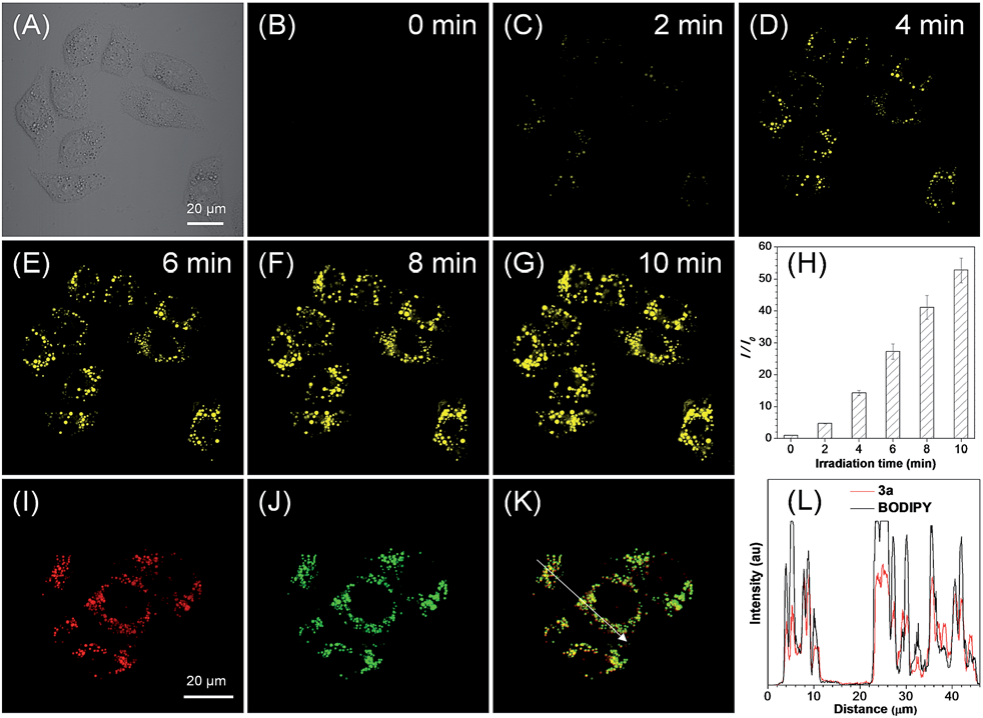
(A–G) Bright-field and fluorescence images of live HeLa cells taken under white light and prolonged irradiation at 405 nm. (H) Plot of fluorescence enhancement (*I*/*I*_0_) of HeLa cells with increasing irradiation time at 405 nm. (I–K) Fluorescence images of HeLa cells stained with the *in situ* generated **3a** after photoactivation (artificial red color) and BODIPY493/503 (green), and the merged image. (L) The intensity profile of ROI lines. [**3a**] = 50 μM.

The cytotoxicities of ethyl mercaptoacetate and the *in situ* generated 2,2′-disulfanediyldiacetate and compounds **3** were then investigated based on MTT assay and no significant decrease in cell viability was observed for both HeLa and MCF-7 cells (Fig. S16–S19†). Because compounds **3** can be *in situ* generated in a quantitative yield, they were then directly used for the cell imaging experiment, which was conducted with HeLa cells as a model cell line. After incubation with the *in situ* generated **3a** for 5 min, a fast light-up fluorescence was observed inside the cells under light irradiation at 405 nm, while almost no fluorescence was observed outside the cells even without washing (Fig. 3A–G). These results suggest that the *in situ* generated **3a** can be quickly taken up by HeLa cells and the photooxidative dehydrogenation reaction can smoothly proceed inside the cells. Through statistical analysis of the intracellular fluorescence intensity, a nearly 53-fold light-up ratio was obtained (Fig. 3H). The PL spectrum of **3a** after photoactivation overlaps well with the “keto” emission spectrum of compound **4a**, which suggests the smooth proceeding of the ESIPT process after photoactivation (Fig. S20†). Furthermore, the co-localization experiment of photoactivated **3a** with lipid dye BODIPY493/503 Green showed a high overlap coefficient of 0.89, which suggests that the probe could selectively accumulate in LDs (Fig. 3I–K and S21A†). Moreover, the intensity profile of photoactivated **3a** and BODIPY493/503 showed a close synchrony for the region of interest (ROI) line across HeLa cells (Fig. 3L), which further verified the specific targeting ability of **3a** for LDs.

**Fig. 4:**
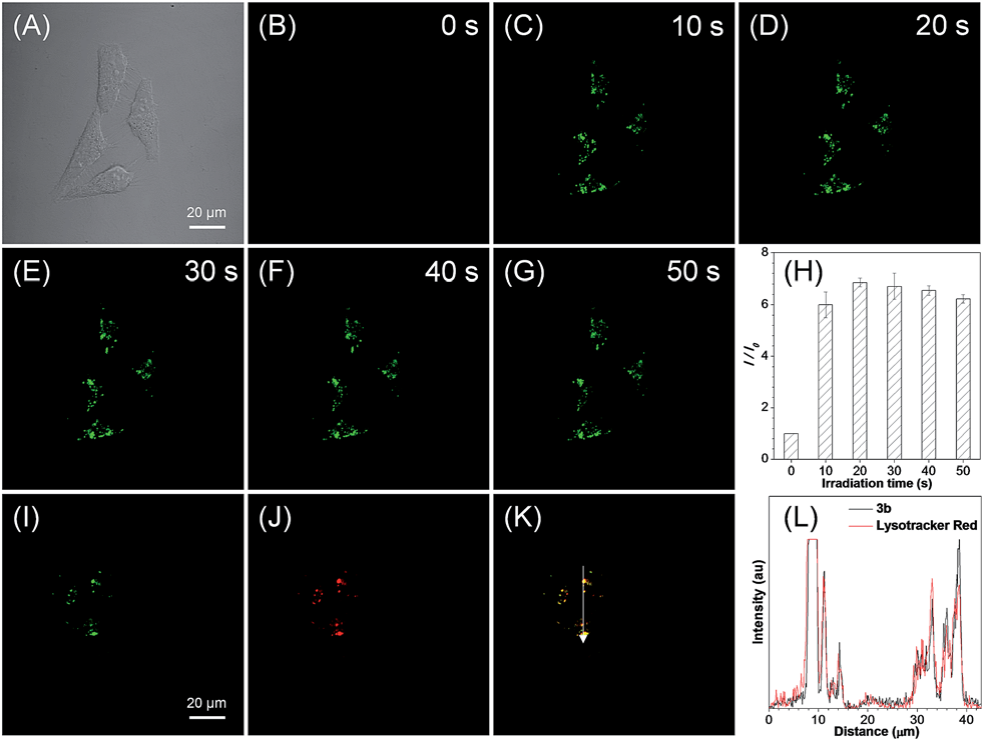
(A–G) Bright-field and fluorescence images of live HeLa cells taken under white light and prolonged irradiation at 405 nm. (H) Plot of fluorescence enhancement (*I*/*I*_0_) of HeLa cells with increasing irradiation time at 405 nm. (I–K) Fluorescence images of HeLa cells stained with the *in situ* generated **3b** after photoactivation (green) and LysoTracker Red (red), and the merged image. (L) The intensity profile of ROI lines. [**3b**] = 20 μM.

For the *in situ* generated compound **3b**, a very fast light-up fluorescence within 20 s and a more than 6-fold light-up ratio was observed for HeLa cells under light irradiation at 405 nm (Fig. 4A–G). The co-staining experiment of photoactivated **3b** with LysoTracker Red showed a high overlap coefficient of 0.86 and an excellent signal synchrony for the ROI line across the cells (Fig. 4I–L and S21B†). These results suggest that the *in situ* generated **3b** with a basic morpholine group could be used for photoactivatable imaging of acidic lysosomes with high specificity. To further verify the organelle-specific imaging ability of the *in situ* generated **3**, the cell imaging experiment was then conducted for MCF-7 cells. A high light-up ratio of 18-fold was observed for **3a** and an excellent overlap coefficient of 0.93 was observed with BODIPY493/503 Green (Fig. S22†). Meanwhile, a 4-fold light-up ratio was observed for **3b** and a high overlap coefficient of 0.88 was observed with LysoTracker Red (Fig. S23†). These results further verified the organelle-specific targeting ability and high photoactivation efficiency of the *in situ* generated compound **3**.

**Fig. 5:**
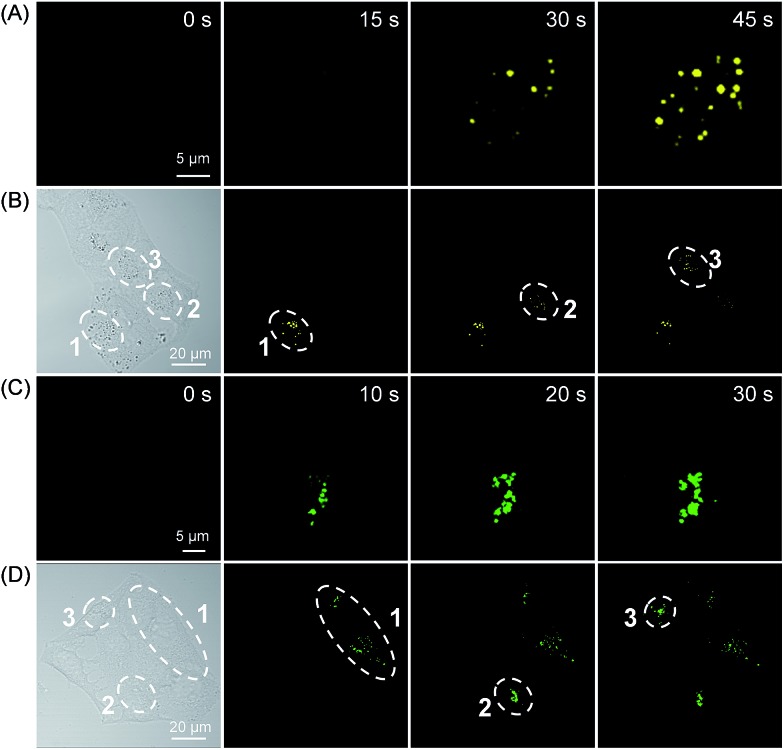
The temporal and spatial photoactivation of LDs (A and B) and lysosomes (C and D) in HeLa cells under two-photon irradiation at 780 nm. [**3a**] = 50 μM; [**3b**] = 20 μM.

To reduce the photo-damage effect of one-photon UV irradiation, the photoactivatable imaging experiment was then investigated under two-photon irradiation at 780 nm. The time-lapse fluorescence images under two-photon irradiation showed a very fast light-up fluorescence within 45 s for both **3a** and **3b** (Fig. 5A and C and S24†). Based on the high spatial imaging resolution under two-photon irradiation, the photoactivation of LDs and lysosomes for selected cells was conducted in a multi-cellular environment. To our satisfaction, the selected cells can be sequentially photoactivated with high spatiotemporal resolution (Fig. 5B and D), which suggests that the photoactivatable probes **3** can be used for organelle study in a complex biological environment.

We also investigated the anti-interfering ability of compounds **3** from chemical oxidants. For both compounds **3a** and **3b** in aqueous solution, almost no fluorescence increase was observed even in the presence of H_2_O_2_ and NaClO (100 μM) for 2 h (Fig. S25†). Moreover, no fluorescence changes were observed for HeLa cells stained with **3** and further treated with H_2_O_2_ or NaClO (100 μM) for 1 h (Fig. S26†). In contrast, a fast light-up fluorescence within 30 s to 5 min was observed under light irradiation at 405 nm, which could thus efficiently preclude the interference of chemical oxidants for the photoactivation process.^14^

## Conclusions

In conclusion, we have developed photoactivatable AIE probes for organelle-specific imaging through *in situ* quantitative generation from easily available disulfide and thiol substrates. The *in situ* generated AIE probes could be directly used for photoactivatable bio-imaging without tedious purification procedures. Based on the adjustable organelle-targeting ability and excellent photoactivation efficiency, high spatiotemporal resolution for photoactivatable imaging of LDs and lysosomes was respectively achieved under both one- and two-photon irradiation. Through avoiding complicated separation steps, the photoactivatable AIE probe could act as an easy-to-use imaging tool and is expected to have broad applications in biological study.

## Conflicts of interest

There are no conflicts to declare.

## Supplementary Material

Supplementary information
