# Supplementary material for: In situ generation of photoactivatable aggregation-induced emission probes for organelle-specific imaging
Source: Chem Sci. 2018 Jun 1;9(26):5730–5. doi: 10.1039/c8sc01887a (PMC6050595; doi:10.1039/c8sc01887a)
Supplement: Supplementary file 1 [file SC-009-C8SC01887A-s001.pdf]

Electronic Supplementary Material (ESI) for Chemical Science.
This journal is © The Royal Society of Chemistry 2018

Supporting Information

In situ generation of photoactivatable aggregation-induced emission probe for organelle-specific imaging

Shiwu Li,[a,b] Xia Ling,[a] Yuhan Lin,[a] Anjun Qin,[a] Meng Gao,*[c] Ben Zhong Tang*[a,d]

[a]Guangdong Innovative Research Team, Center for Aggregation-Induced Emission, State Key Laboratory of Luminescent Materials & Devices, South China University of Technology, Guangzhou 510640, China.

[b]School of Medicine, South China University of Technology, Guangzhou 510006, China.

[c]National Engineering Research Center for Tissue Restoration and Reconstruction, South China University of Technology, Guangzhou 510006, China.

[d]Department of Chemistry and Hong Kong Branch of Chinese National Engineering Research Center for Tissue Restoration and Reconstruction, The Hong Kong University of Science & Technology, Clear Water Bay, Kowloon, Hong Kong, China.

*Corresponding Author: msmgao@scut.edu.cn; tangbenz@ust.hk

Table of Contents

Materials and chemicals S3

Equipment and methods S3

Scheme S1. Synthetic routes of compounds **1**, **3**, and **4** S9

Table S1 Photophysical properties of compounds **4** S16

Fig. S1 ^{1}H and ^{13}C NMR spectra of **1a** S10

Fig. S2 ^{1}H and ^{13}C NMR spectra of **1b** S11

Fig. S3 ^{1}H and ^{13}C NMR spectra of **3a** S12

Fig. S4 ^{1}H and ^{13}C NMR spectra of **3b** S13

Fig. S5 ^{1}H and ^{13}C NMR spectra of **4a** S14

Fig. S6 ^{1}H and ^{13}C NMR spectra of **4b** S15

Fig. S7 The UV-Vis and PL spectra of **4** S16

Fig. S8 The ^{1}H NMR stacking spectra of ethyl mercaptoacetate, "**1** + ethyl mercaptoacetate" and 2,2'-disulfanediyldiacetate S17

Fig. S9 The UV-Vis spectra of **1**, "**1** + ethyl mercaptoacetate", and **3** S18

Fig. S10 The ^{1}H NMR stacking spectra of **1b**, "**1b** + ethyl mercaptoacetate", and **3b** S18

Fig. S11 The HPLC spectra showing the complete in situ transformation S19

Fig. S12 The ^{1}H NMR spectra of the mixture of **1a** and ethyl mercaptoacetate under UV irradiation S19

Fig. S13 The UV-Vis and PL spectra of in situ generated **3b** under UV irradiation S20

Fig. S14 The bright field and fluorescence images of **3b** in solid state under UV irradiation S20

Fig. S15 The ^{1}H NMR spectra of the mixture of **1b** and ethyl mercaptoacetate under UV irradiation S21

Fig. S16 The cell viabilities of HeLa and MCF-7 cells treated with ethyl mercaptoacetate S21

Fig. S17 The cell viabilities of HeLa and MCF-7 cells treated with 2,2'-disulfanediyldiacetate S22

Fig. S18 The cell viabilities of HeLa and MCF-7 cells treated with in situ generated **3a** S22

Fig. S19 The cell viabilities of HeLa and MCF-7 cells treated with in situ generated **3b** S22

Fig. S20 The fluorescence images and PL spectra of HeLa cells treated with **3** and after photoactivation S23

Fig. S21 The intensity correlation plots for co-staining experiment S23

Fig. S22 The bright field and fluorescence images of MCF-7 cells stained with **3a** and co-stained with BODIPY493/503 Green S24

Fig. S23 The bright field and fluorescence images of MCF-7 cells stained with **3b** and co-stained with LysoTracker Red S24

Fig. S24 Plots of fluorescence enhancement (I/I_0) of HeLa cells under two-photon irradiation S25

Fig. S25 The PL spectra measurement for **3a** treated with chemical oxidants S25

Fig. S26 The fluorescence images of HeLa and MCF-7 cells treated with **3** and chemical oxidants S26

References S26

Materials and chemicals

2-hydroxy-5-(morpholinomethyl) benzaldehyde, 2,2'-disulfidediyldianiline, and 2-(2,3-dihydrobenzo[*d*]thiazol-2-yl)-4-methoxyphenol were prepared according to the literature method.[1-4] Morpholine, 2-Aminobenzenethiol and 2-hydroxy-5-methoxybenzaldehyde were purchased from Energy Chemical; $CDCl_3$ and DMSO-d_6 were purchased from Sigma-Aldrich; H_2O_2 (30 wt.%), HOAc, K_2CO_3, MeOH and other solvent were purchased from Guangzhou Chemical Reagent Factory without further purification. THF was distilled from sodium under dry nitrogen prior to use.

Dulbecco's Modified Essential Medium (DMEM) and Hanks' Balanced Salt Solution (HBSS) buffer were purchased from Gibco (Life Technologies). Ultra pure water was supplied by Milli-Q Plus System (Millipore Corporation, United States). Phosphate buffered saline (PBS), fetal bovine serum (FBS), penicillin, streptomycin, BODIPY 493/503, and LysoTracker Red were purchased from Thermo Fisher Scientific. The HeLa and MCF-7 cell lines were purchased from the Cell Resource Center, Peking Union Medical College.

Equipment and methods

UV-Vis absorption spectra were measured on a Shimadzu UV-2600 spectrophotometer, medium scanning rate, and quartz cuvettes of 1 cm path length. Photoluminescence spectra were recorded on a Horiba Fluoromax-4 spectrofluorometer. The absolute fluorescence quantum yield was measured using a Hamamatsu quantum yield spectrometer C11347 Quantaurus_QY. The fluorescence lifetime was measured using a Hamamatsu Compact Fluorescence Lifetime Spectrometer C11367. ^{1}H and ^{13}C NMR spectra were measured on a Bruker AV 500 NMR spectrometer. High resolution mass spectra (HRMS) were recorded on a Bruker maxis impact mass spectrometer operated in MALDI-TOF or ESI model. Confocal lasing scanning microscopic (CLSM) images

were obtained on the confocal microscope (Zeiss Laser Scanning Confocal Microscope; LSM710). Two-photon excited fluorescence images were obtained on the multiphoton laser scanning microscope (Olympus FV1200MPE). Fluorescence images were obtained on the fluorescence optical microscope (Zeiss Axio Vert.A1). Automated cell counter (Countess II) was employed for cell counting. The cell viability analysis for estimating cytotoxicity was collected using a microplate reader (Tecan Infinite M200 PRO) at a wavelength of 570 nm. The HPLC profiles were acquired on an Waters e2695 instrument with a SunFire C18 column (4.6 mm × 250 mm, 5 μm) and UV–Visible detector (Waters 2489) set at 315 nm, acetonitrile was used as the eluent with the flow rate of 0.6 mL min^{-1}.

Synthesis of 2,2'-disulfanediyldianiline. 2-Aminobenzenethiol (500 mg, 4 mmol) was first dissolved in methanol (5 mL), H_2O_2 (30 wt.%, 0.5 mL) was then added with drop-wise. The mixture was further stirred at room temperature for 30 min. After completion of the reaction, the mixture was filtered and the precipitation was washed by ethanol to afford a yellow solid (445 mg, yield 90%).

Synthesis of 2-hydroxy-5-(morpholinomethyl)benzaldehyde. 5-(Chloromethyl)-2-hydroxybenzaldehyde (850 mg, 5.0 mmol) was first dissolved in MeCN (10 mL), morpholine (479 μL, 5.5 mmol) and K_2CO_3 (759 mg, 5.5 mmol) were then added into the mixture, which was stirred at room temperature for 0.5 h. After completion of the reaction, the K_2CO_3 was removed by filtration. The filtrate solution was dried by evaporation under vacuum and further extracted with dichloromethane (40 mL × 3). The extracts were washed with brine, dried over anhydrous $MgSO_4$, and concentrated under reduced pressure. The residue was further separated by column chromatography

(petroleum ether : ethyl acetate = 7 : 3) to afford a white solid (464 mg, yield 42%). ^{1}H NMR ($CDCl_3$, 500 MHz) : 10.96 (br s, 1H), 9.90 (d, J = 0.5 Hz, 1H), 7.53-7.50 (m, 2H), 6.96 (d, J = 8.5 Hz, 1H), 3.72 (t, J = 4.5 Hz, 4H), 3.48 (s, 2H), 2.46 (br s, 4H).

Synthesis of 2,2'-((1*E*,1'*E*)-((disulfanediylbis(2,1-phenylene))bis(azanylylidene))bis(methanylylidene))bis(4-methoxyphenol) (1a). 2,2'-Disulfanediyldianiline (248 mg, 1.0 mmol) was first dissolved in ethanol (10 mL), 2-hydroxy-5-methoxybenzaldehyde (380 mg, 2.5 mmol) and a drop of HOAc were then added. The mixture further reacted under reflux and nitrogen protection for 1 h. After completion of the reaction, the mixture was filtered and the precipitation was washed by ethanol to afford a yellow solid (495 mg, yield 96%). ^{1}H NMR (DMSO-d_6, 500 MHz) : 11.98 (s, 2H), 9.00 (s, 2H), 7.59 (dd, J_1 = 8.0 Hz, J_2 = 1.5 Hz, 2H), 7.49 (dd, J_1 = 8.0 Hz, J_2 = 1.0 Hz, 2H), 7.37 (td, J_1 = 7.5 Hz, J_2 = 1.5 Hz, 2H), 7.32-7.29(m, 4H), 7.10 (dd, J_1 = 9.0 Hz, J_2 = 3.0 Hz, 2H), 6.96 (d, J = 9.0 Hz, 2H), 3.77 (s, 6H) ; ^{13}C NMR (DMSO-d_6, 125 MHz) : 163.0, 154.3, 152.0, 146.1, 130.4, 128.1, 127.9, 126.0, 121.2, 119.3, 118.3, 117.6, 115.1, 55.6. HRMS (MALDI-TOF): m/z $[M + H]^+$ calcd. for $C_{28}H_{25}N_2O_4S_2$, 517.1250, found, 517.1301.

Synthesis of 2,2'-((1E,1'E)-((disulfanediylbis(2,1-phenylene))bis(azanylylidene))bis(methanylylidene))bis(4-(morpholinomethyl)phenol (1b). 2,2'-Disulfanediyldianiline (62 mg, 0.3 mmol) was first dissolved in ethanol (5 mL), 2-hydroxy-5-(morpholinomethyl)benzaldehyde (130 mg, 0.6 mmol) and a drop of HOAc were then added. The mixture was further stirred under nitrogen protection for 1 h. After completion of the reaction, the mixture was

filtered and the precipitation was washed by ethanol to afford a light yellow solid (152 mg, yield 78%). ^{1}H NMR (DMSO-d_6, 500 MHz): δ 12.51 (s, 2H), 9.03 (s, 2H), 7.65 (d, J = 1.5 Hz, 2H), 7.58 (J_1 = 7.5 Hz, J_2 = 1.5 Hz, 2H), 7.51(dd, J_1 = 7.5 Hz, J_2 = 1.0 Hz, 2H), 7.41-7.35(m, 4H), 7.31-7.28(m, 2H), 6.97 (d, J = 8.5 Hz, 2H), 3.58 (t, J = 4.5 Hz, 8H), 3.44 (s, 4H), 2.37 (s, 8H); ^{13}C NMR (DMSO-d_6, 125 MHz): 163.6, 159.2, 146.1, 134.7, 132.9, 130.3, 128.6, 128.2, 127.9, 126.1, 119.0, 118.5, 116.6, 66.2, 61.6, 53.1. HRMS (ESI): m/z [M + H]$^+$ calcd. for $C_{36}H_{39}N_4O_4S_2$, 655.2407, found, 655.2415.

Synthesis of compound 2-(2,3-dihydrobenzo[d]thiazol-2-yl)-4-methoxyphenol (3a). 2-Aminobenzenethiol (625 mg, 5.0 mmol) and 2-hydroxy-5-methoxybenzaldehyde (760 mg, 5.0 mmol) were dissolved in methanol (10 mL) under nitrogen protection. The mixture was stirred at room temperature for 20 min. After completion of the reaction, the mixture was filtered and the precipitation was washed by methanol to afford a white solid (776 mg, yield 60%). ^{1}H NMR (DMSO-d_6, 500 MHz): δ 9.35 (s, 1H), 6.95-6.93 (m, 2H), 6.86 (td, J_1 = 9.5 Hz, J_2 = 1.5 Hz, 1H), 6.78-6.65 (m, 4H), 6.57 (td, J_1 = 9.5 Hz, J_2 = 1.5 Hz, 1H), 6.43 (d, J = 3.0 Hz, 1H), 3.62 (s, 3H); ^{13}C NMR (DMSO-d_6, 125 MHz): 152.0, 147.7, 147.3, 130.3, 125.2, 125.1, 121.2, 118.6, 115.5, 113.2, 111.9, 108.7, 63.2, 55.3.

Synthesis of compound 2-(2,3-dihydrobenzo[d]thiazol-2-yl)-4-(morpholinomethyl)phenol (3b). 2-Aminobenzenethiol (125 mg, 1.0 mmol) and 2-hydroxy-5-(morpholinomethyl)benzaldehyde (221 mg, 1.0 mmol) were dissolved in methanol (5 mL) under nitrogen protection. The mixture was stirred at room temperature for 20 min. After completion of the reaction, the mixture was evaporated

under reduced pressure and the residue was recrystallized with methanol. After further drying under vacuum, a white solid was obtained (171 mg, yield 52%). ^{1}H NMR (DMSO-d_6, 500 MHz): δ 9.75 (s, 1H), 7.37 (d, J = 2.0 Hz, 1H), 7.02 (dd, J_1 = 8.5 Hz, J_2 = 2.0 Hz, 1H), 6.93 (d, J = 7.5 Hz, 1H), 6.86 (td, J_1 = 7.5 Hz, J_2 = 1.0 Hz, 1H), 6.76 (d, J = 8.0 Hz, 2H), 6.65 (d, J = 7.5 Hz, 1H), 6.56 (td, J_1 = 7.5 Hz, J_2 = 1.0 Hz, 1H), 6.50 (d, J = 2.5 Hz, 1H), 3.51 (t, J = 4.5 Hz, 4H), 3.30 (d, J = 5.0 Hz, 2H), 2.27 (s, 4H); ^{13}C NMR (DMSO-d_6, 125 MHz): 152.7, 147.8, 129.3, 128.9, 127.7, 126.7, 125.3, 125.1, 121.1, 118.4, 114.6, 108.6, 66.1, 63.3, 62.2, 53.0.

Synthesis of 2-(benzo[d]thiazol-2-yl)-4-methoxyphenol (4a). 2-Aminobenzenethiol (250 mg, 2.0 mmol) and 2-hydroxy-5-methoxybenzaldehyde (304 mg, 2.0 mmol) were first dissolved in methanol (5 mL), conc. HCl (37 wt.%, 169 μL) and H_2O_2 (30 wt.%, 188 μL) were then added in to the mixture, which was further stirred at room temperature for 2 h. After completion of the reaction, the precipitation was filtered and washed by methanol (10 mL × 2). After further drying under vacuum, a white solid was obtained (493 mg, yield 96%). ^{1}H NMR (DMSO-d_6, 500 MHz): δ 11.03 (s, 1H), 8.14 (d, J = 8.0 Hz, 1H), 8.07 (d, J = 8.0 Hz, 1H), 7.72 (d, J = 3.0 Hz, 1H), 7.54 (td, J_1 = 8.0 Hz, J_2 = 1.0 Hz, 1H), 7.44 (td, J_1 = 8.0 Hz, J_2 = 1.0 Hz, 1H), 7.06-7.01 (m, 2H), 3.81 (s, 3H); ^{13}C NMR (DMSO-d_6, 125 MHz): 164.3, 152.2, 151.4, 150.4, 134.7, 126.4, 125.0, 122.2, 121.9, 119.7, 118.7, 118.0, 111.1, 55.6. HRMS (ESI): m/z $[M + H]^+$ calcd. for $C_{14}H_{12}NO_2S$, 258.0583, found, 258.0581.

Synthesis of 2-(benzo[d]thiazol-2-yl)-4-(morpholinomethyl)phenol (4b). 2-Aminobenzenethiol (63 mg, 0.5 mmol) and 2-hydroxy-5-

(morpholinomethyl)benzaldehyd (111 mg, 0.5 mmol) were dissolved in methanol (5 mL), conc. HCl (37 wt.%, 42 μL) and H_2O_2 (30 wt.%, 47 μL) were then added. The mixture was stirred at room temperature for 2 h. After completion of the reaction, the solvent was evaporated under reduced pressure and the residue was purified by repeated washing with THF. After filtration and further drying under vacuum, a grey solid was obtained (124 mg, yield 76%). ^{1}H NMR (DMSO-d_6, 500 MHz): δ 12.04 (br s, 1H), 8.12 (d, J = 8.0 Hz, 2H), 8.04 (d, J = 8.0 Hz, 1H), 7.52 (t, J = 7.5 Hz, 1H), 7.42 (t, J = 7.5 Hz, 1H), 7.30 (d, J = 8.0 Hz, 1H), 7.03 (d, J = 8.5 Hz, 1H), 3.58 (s, 4H), 3.45 (s, 2H), 2.38 (s, 4H); ^{13}C NMR (DMSO-d_6, 125 MHz): 165.1, 151.6, 135.1, 133.1, 128.5, 125.8, 124.2, 123.9, 121.7, 121.4, 118.5, 117.8, 66.2, 62.3, 53.1. HRMS (ESI): m/z $[M + H]^+$ calcd. for $C_{18}H_{19}N_2O_2S$, 327.1162, found, 327.1157.

Cell viability measurement

The cells were seeded in a 96-well plate at a density of 2×10^5 cells/mL. After 24 h of culture, different concentrations of the in situ generated compound **3** were added and further incubated for 24 h. The sample and control wells were washed twice with PBS buffer and added with freshly prepared MTT solution (0.5 mg/mL, 100 μL). After incubation at 37 °C for 4 h, the MTT solution was removed and washed twice with PBS buffer. DMSO (100 μL) was then added into each well and the plate was gently shaken for 10 min at room temperature to dissolve all the precipitates formed. The absorbance of sample and control wells at 570 nm was then measured by a microplate Reader. Cell viability was then calculated by the ratio of the absorbance of sample wells to control cells.

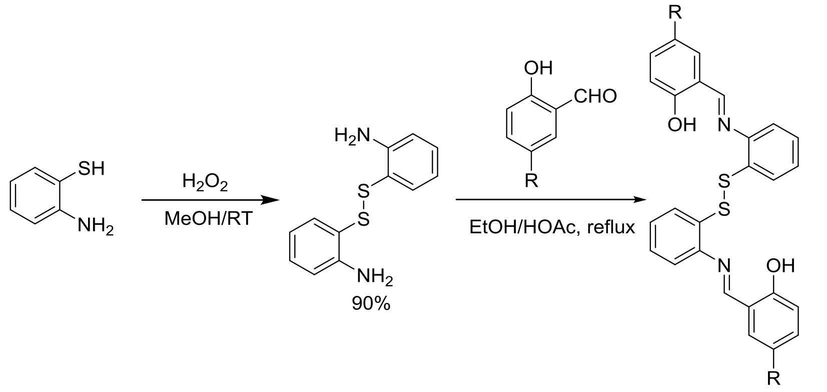

1a, R = OMe, 96%;
1b, R = morpholinomethyl, 78%;

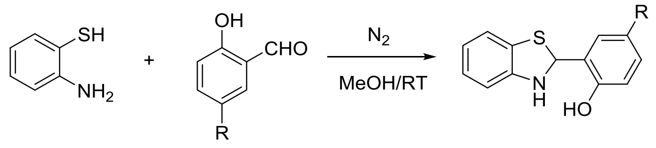

3a, R = OMe, 60%;
3b, R = morpholinomethyl, 52%;

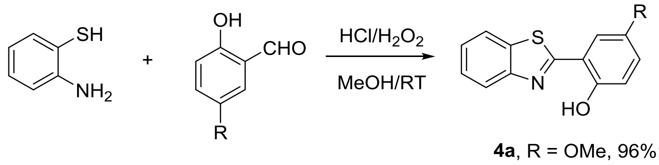

4a, R = OMe, 96%;
4b, R = morpholinomethyl, 76%;

Scheme S1 Synthetic routes of compounds **1**, **3**, and **4**.

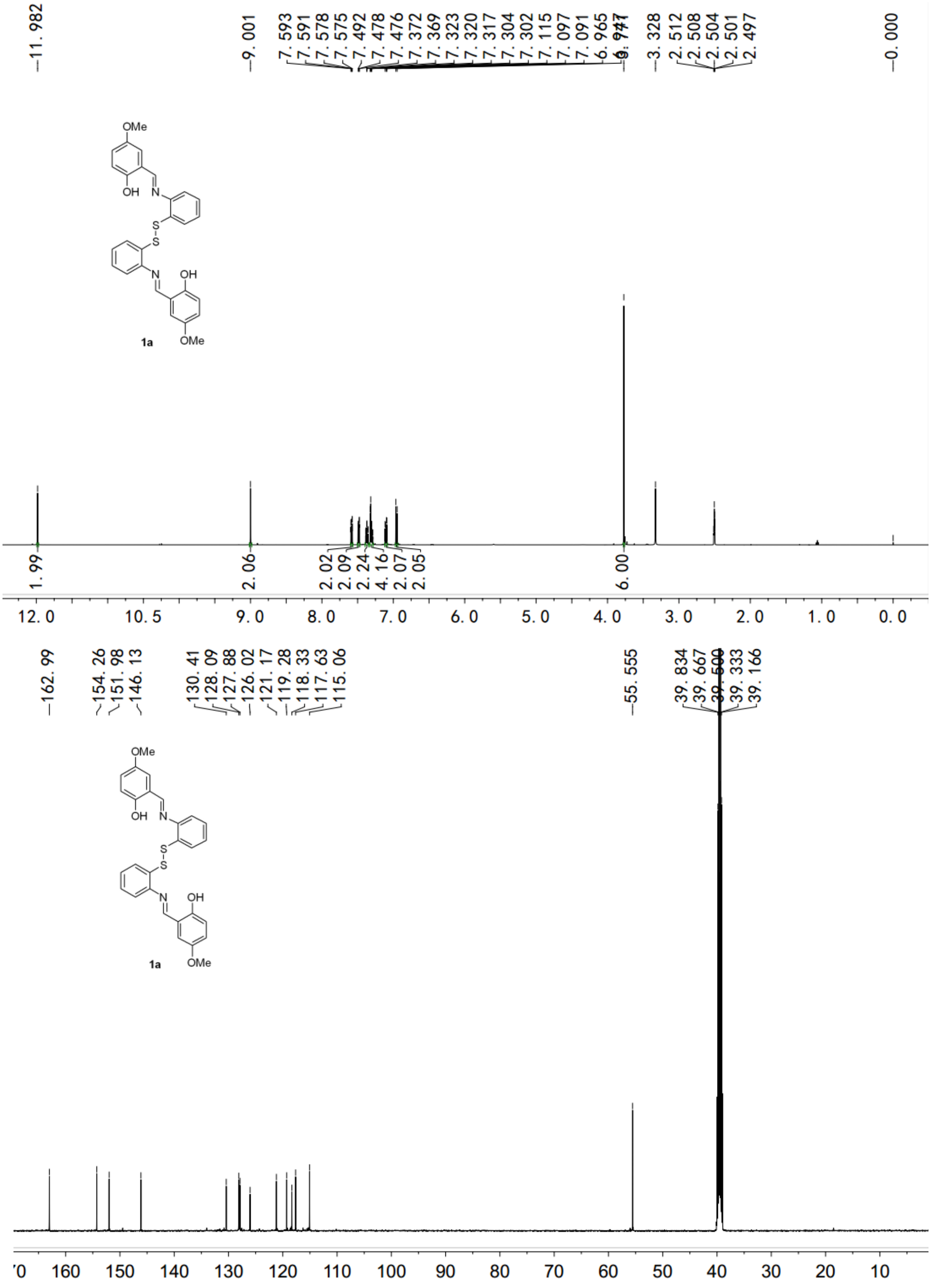

Fig. S1 ^{1}H and ^{13}C NMR spectra of **1a**.

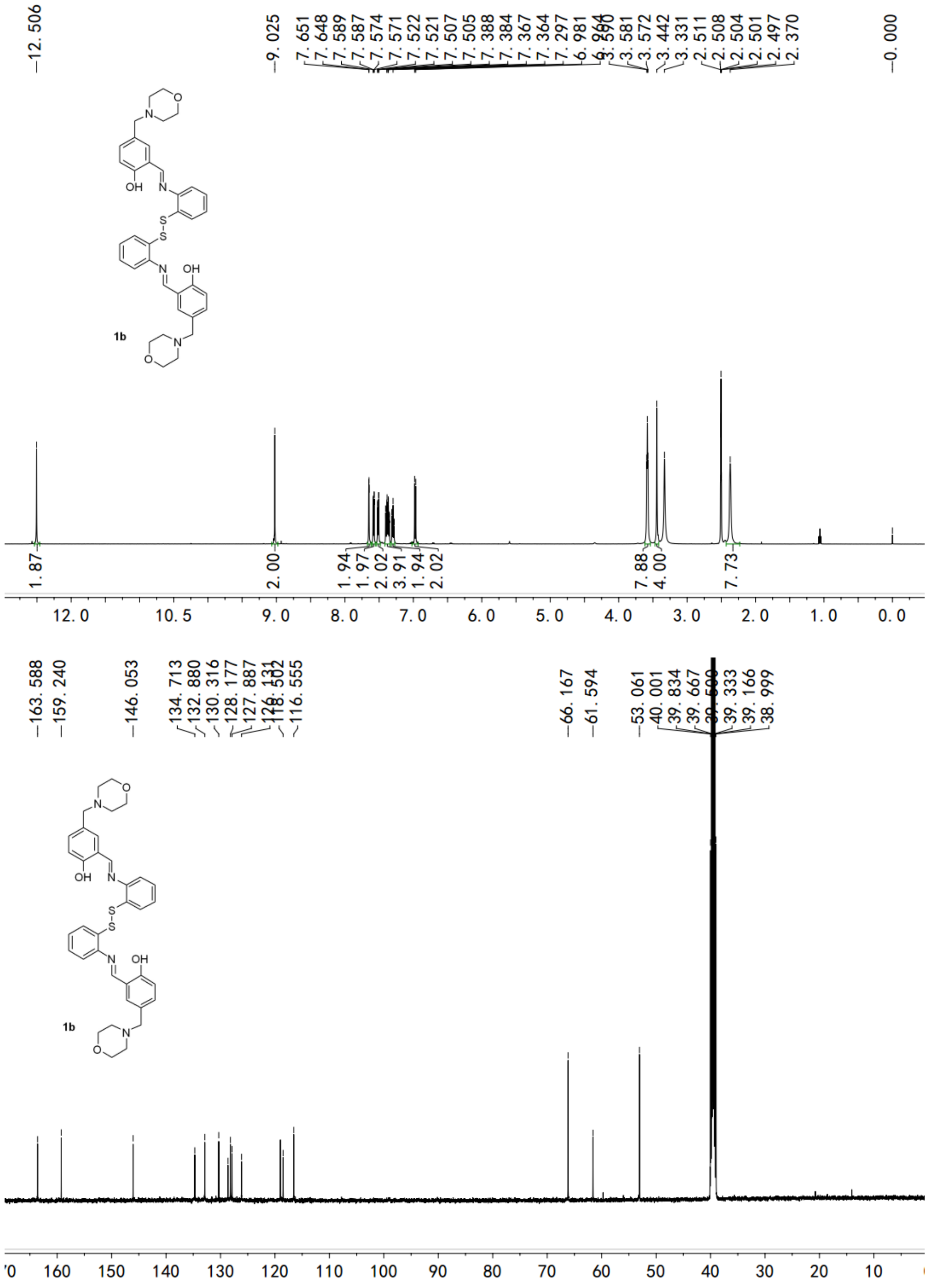

Fig. S2 ^{1}H and ^{13}C NMR spectra of **1b**.

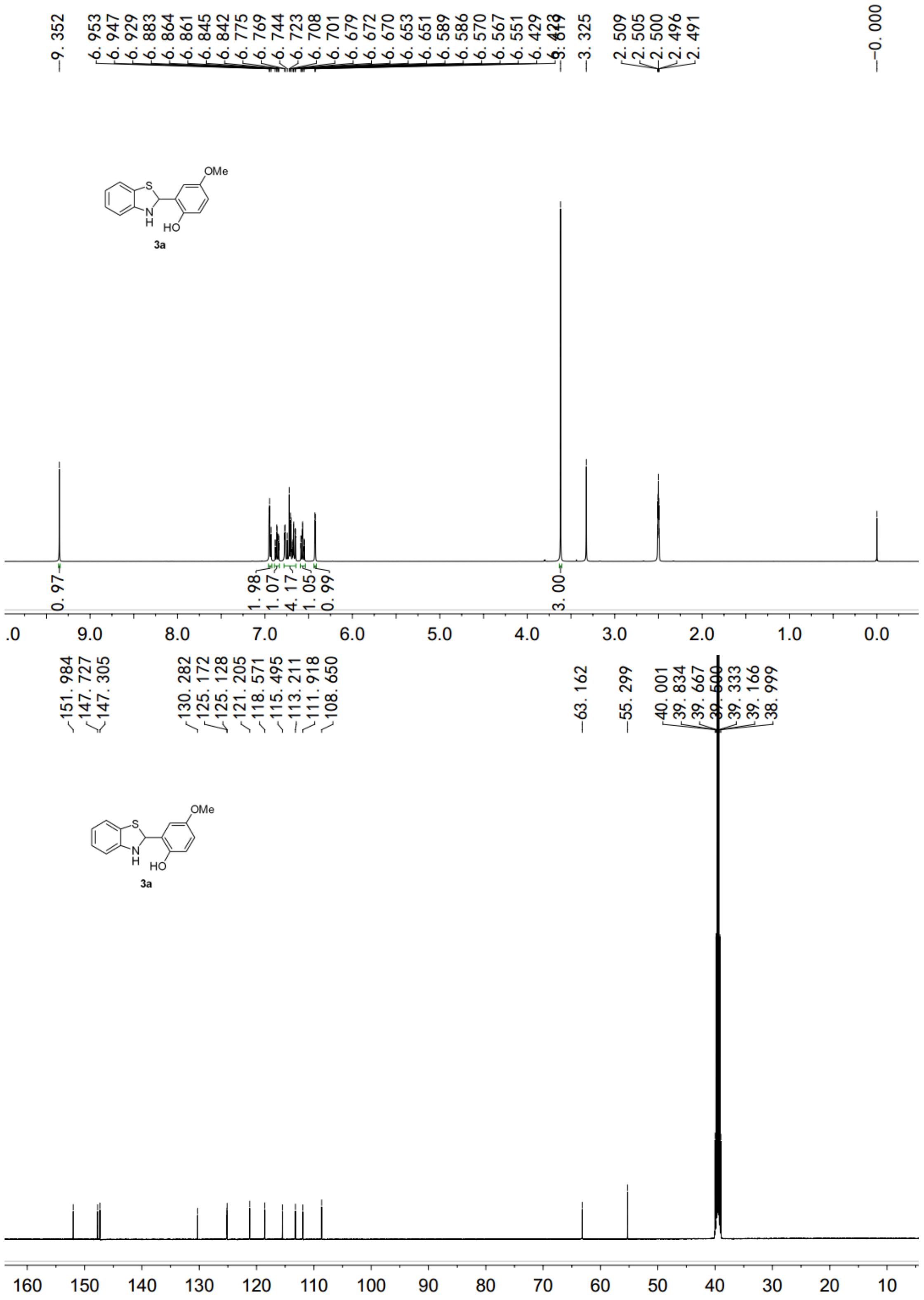

Fig. S3 ^{1}H and ^{13}C NMR spectra of **3a**.

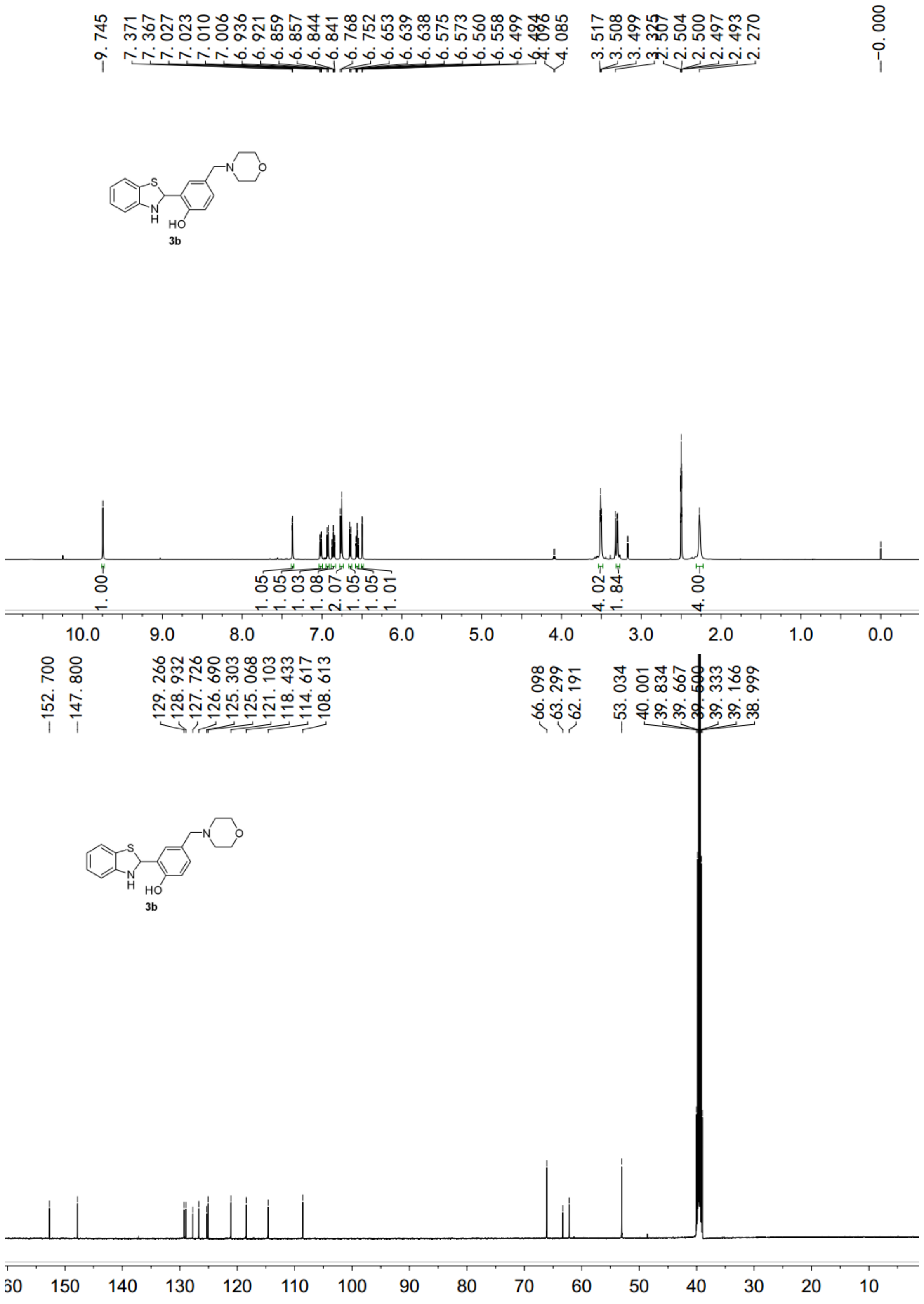

Fig. S4 ^{1}H and ^{13}C NMR spectra of **3b**.

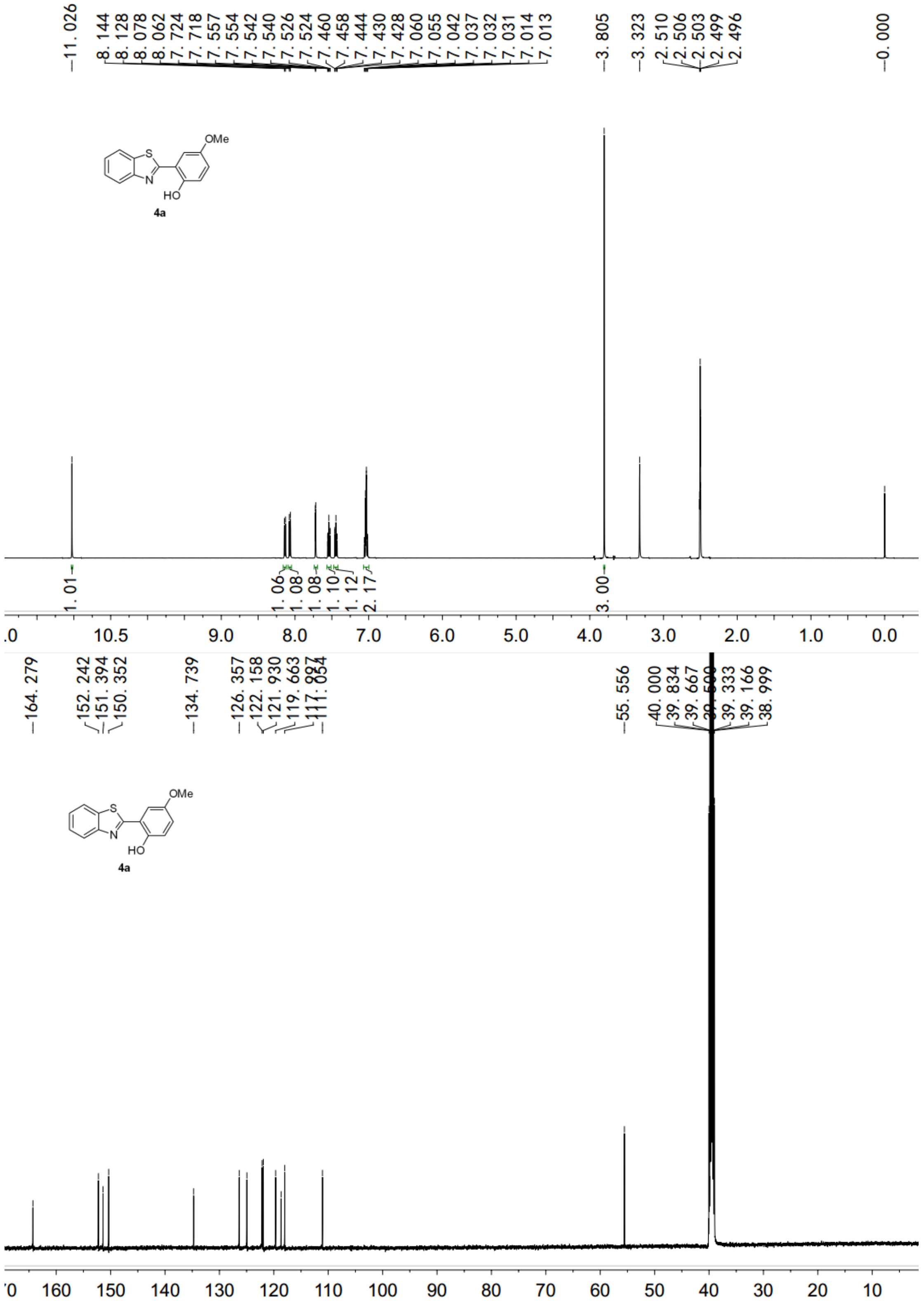

Fig. S5 ^{1}H and ^{13}C NMR spectra of **4a**.

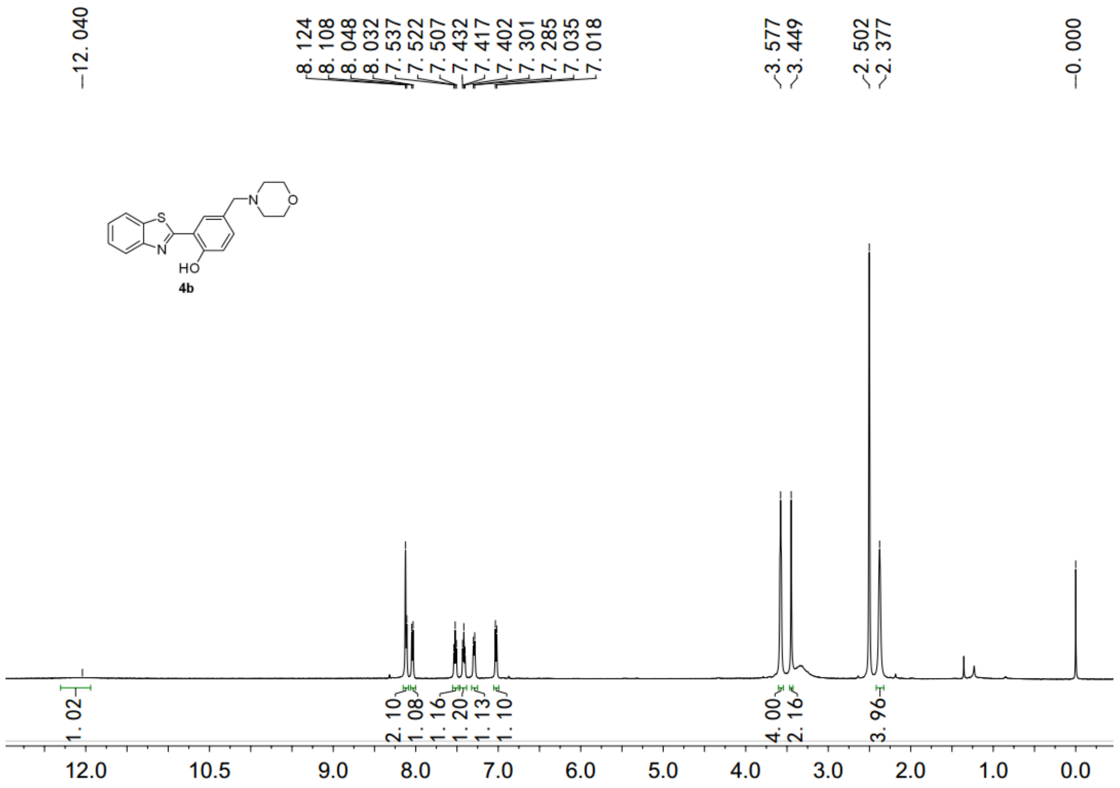

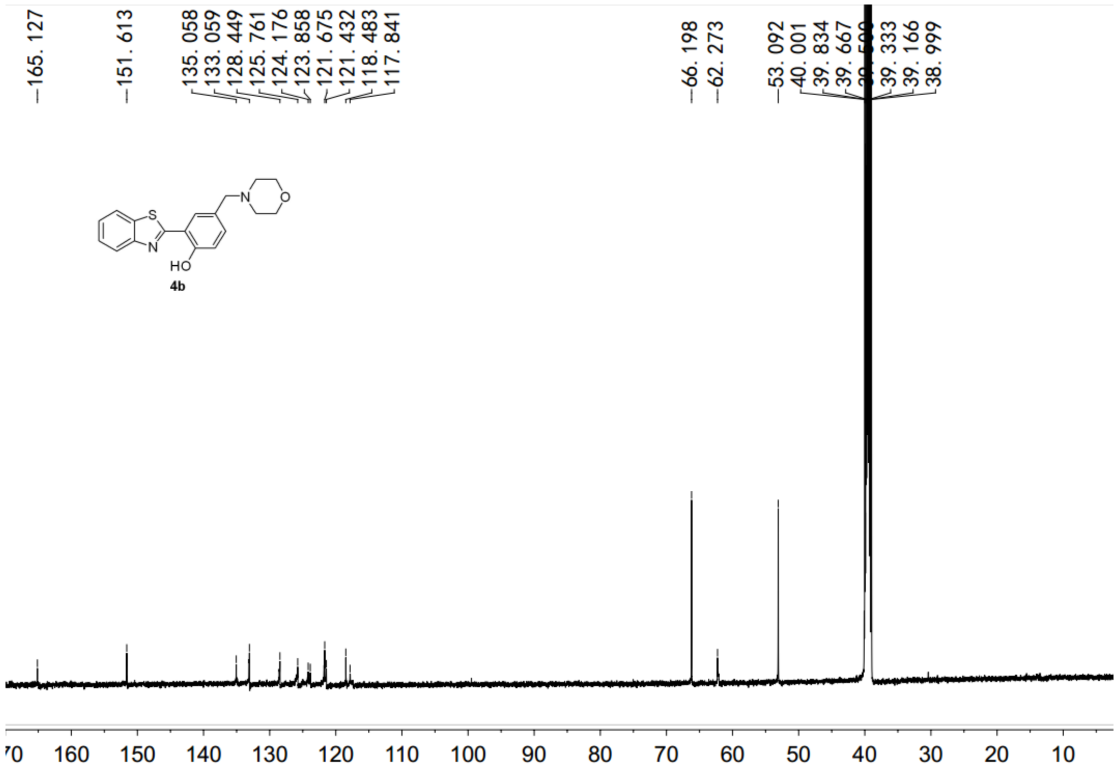

Fig. S6 ^{1}H and ^{13}C NMR spectra of **4b**.

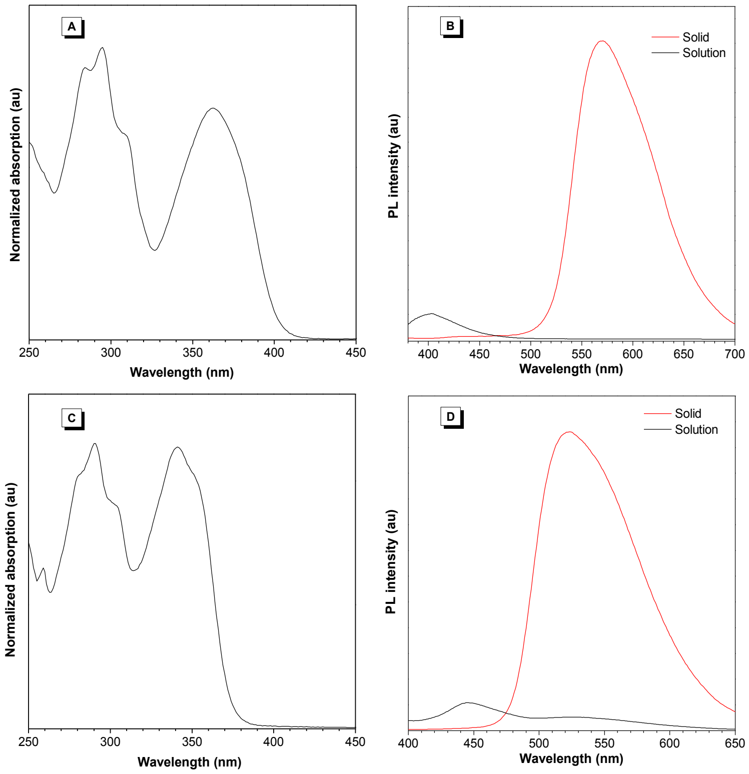

Fig. S7 (A) The normalized UV−Vis absorption spectra of **4a** in THF solution. (B) The PL spectra of **4a** in THF solution and at solid state; [**4a**] = 10 μM; λ_{ex} = 362 nm. (C) The normalized UV−Vis absorption spectra of **4b** in THF solution. (D) The PL spectra of **4b** in THF solution and at solid state; [**4b**] = 10 μM; λ_{ex} = 341 nm.

Table S1. Photophysical properties of compound **4** in THF solution (Soln) and solid (Solid) states.

**4**	**Soln**[a)]						**Solid**[b)]				
	λ_{ab} [nm][c)]	λ_{em} [nm][d)]	Φ_F [%][e)]	τ (ns)[f)]	k_r [10^7 S^{-1}][g)]	k_{nr} [10^8 S^{-1}][h)]	λ_{em} [nm][d]	Φ_F [%][e)]	τ (ns)[f)]	k_r [10^7 S^{-1}][g)]	k_{nr} [10^8 S^{-1}][h)]
**4a**	362	404	1.2	0.94	1.28	10.5	570	40.1	4.88	8.22	1.23
**4b**	341	446	1.1	1.56	0.71	6.34	523	28.4	6.39	4.44	1.12

a) In THF solution with a concentration of 10^{-5} M; b) solid powder; c) Maximum absorption wavelength; d) Maximum emission wavelength; e) Absolute quantum yield; f) Average fluorescence lifetime; g) Radiative relaxation rate $k_r = \Phi/\tau$; h) Non-radiative relaxation rate $k_{nr} = (1-\Phi)/\tau$.

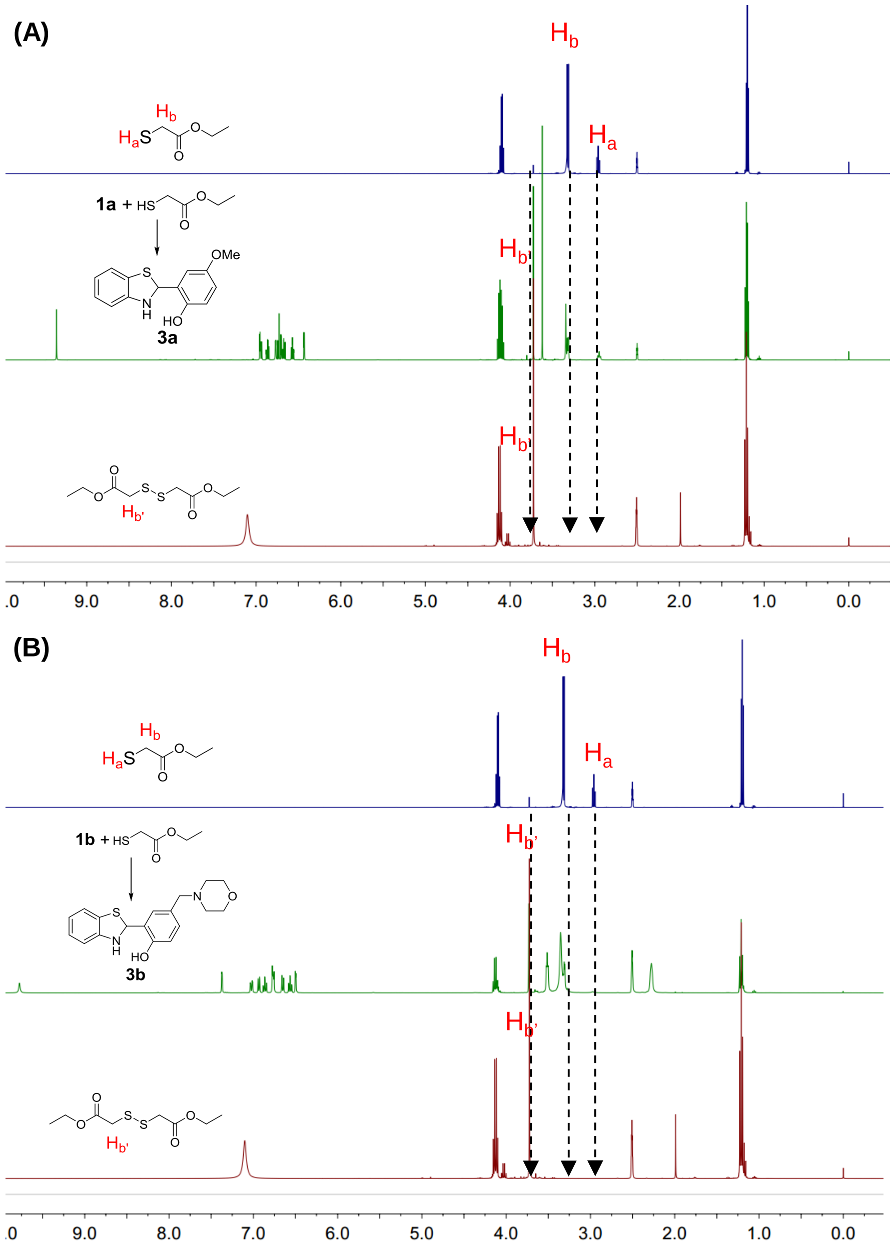

Fig. S8 The ^{1}H NMR stacking spectra: (A) ethyl mercaptoacetate, "**1a** + ethyl mercaptoacetate", and 2,2'-disulfanediyldiacetate; (B) ethyl mercaptoacetate, "**1b** + ethyl mercaptoacetate", and 2,2'-disulfanediyldiacetate.

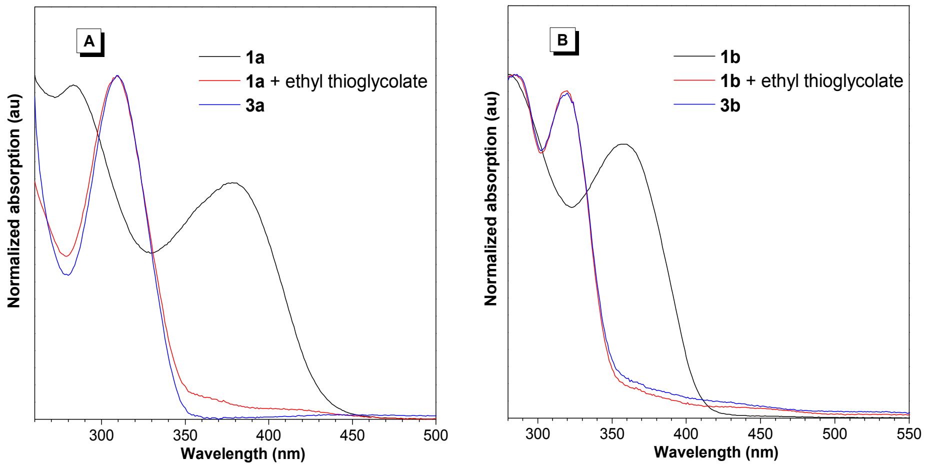

Fig. S9 The normalized UV-Vis spectra of (A) **1a**, "**1a** + ethyl mercaptoacetate", and **3a**; (B) **1b**, "**1b** + ethyl mercaptoacetate", and **3b**. [**1a**] = [**1b**] = 10 μM; [**3a**] = [**3b**] = 20 μM.

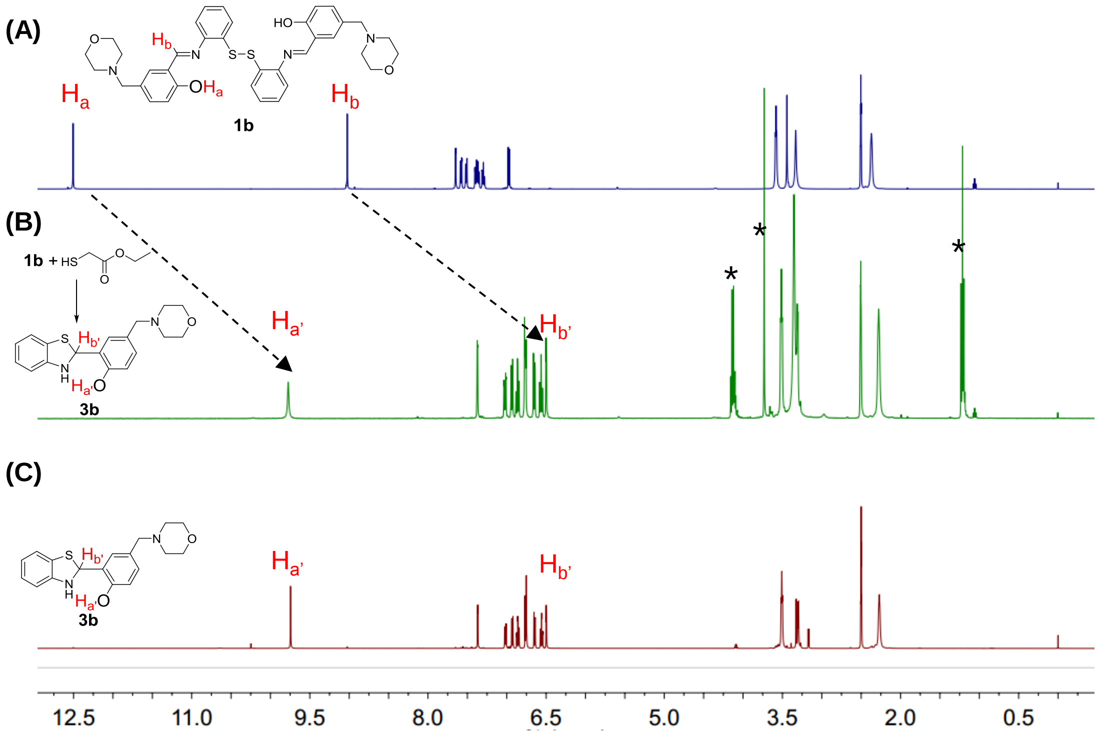

Fig. S10 The ^{1}H NMR stacking spectra of (A) **1b**, (B) "**1b** + ethyl mercaptoacetate", and (C) **3b**. (* represents diethyl 2,2'-disulfanediyldiacetate)

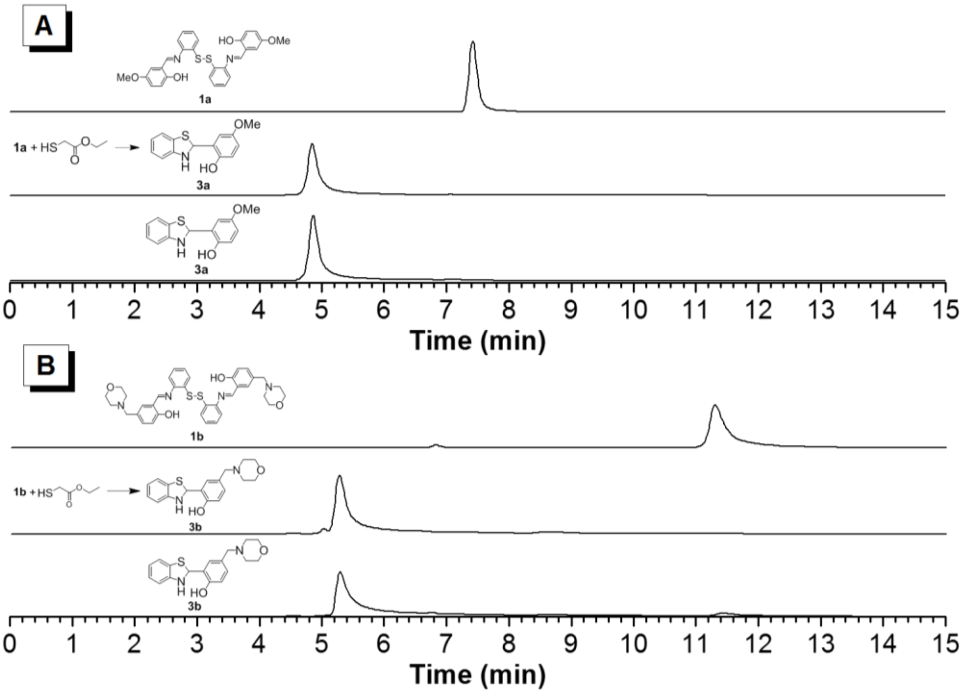

Fig. S11 The HPLC spectra showing the complete in situ transformation of disulfide and thiol substrates into 2-(2-hydroxyphenyl)-benzothiazolines: (A) **1a**, "**1a** + ethyl mercaptoacetate", and purified **3a**; (B) **1b**, "**1b** + ethyl mercaptoacetate", and purified **3b**.

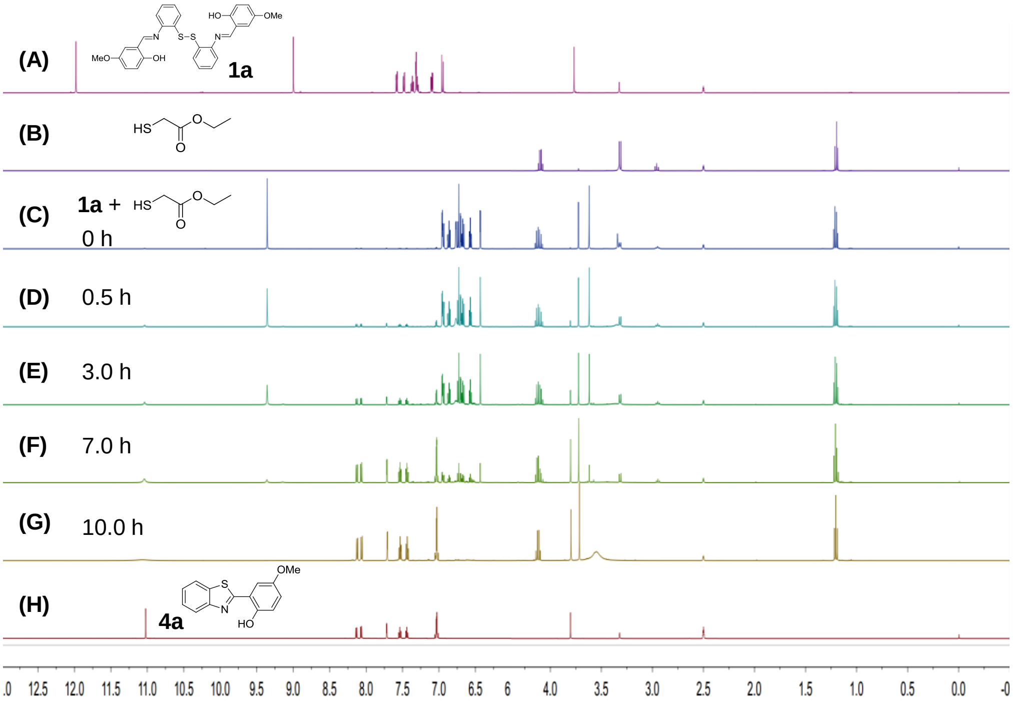

Fig. S12 The ^{1}H NMR stacking spectra for in situ monitoring of the reaction between **1a** and ethyl mercaptoacetate under UV irradiation (365 nm).

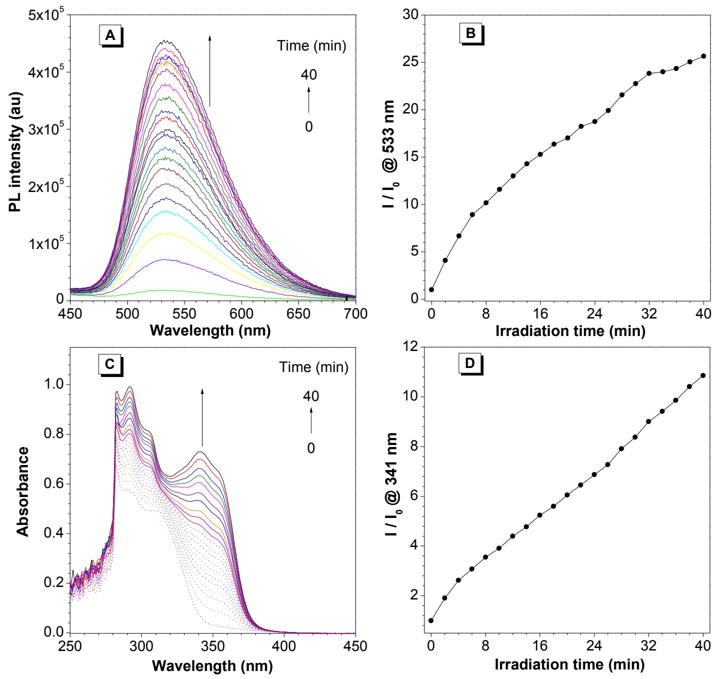

Fig. S13 (A) The PL spectra of in situ generated **3b** in DMSO/toluene mixture (v/v, 1/99) under irradiation at 365 nm for 0–20 min. (B) Plot of relative PL intensity I/I_0 at 533 nm versus the irradiation time. (C) The UV-Vis spectra of in situ generated **3b** in DMSO/toluene mixture (v/v, 1/99) under irradiation at 365 nm for 0–20 min. (D) Plot of relative UV-Vis absorption intensity I/I_0 at 341 nm versus the irradiation time. [**3b**] = 100 μM.

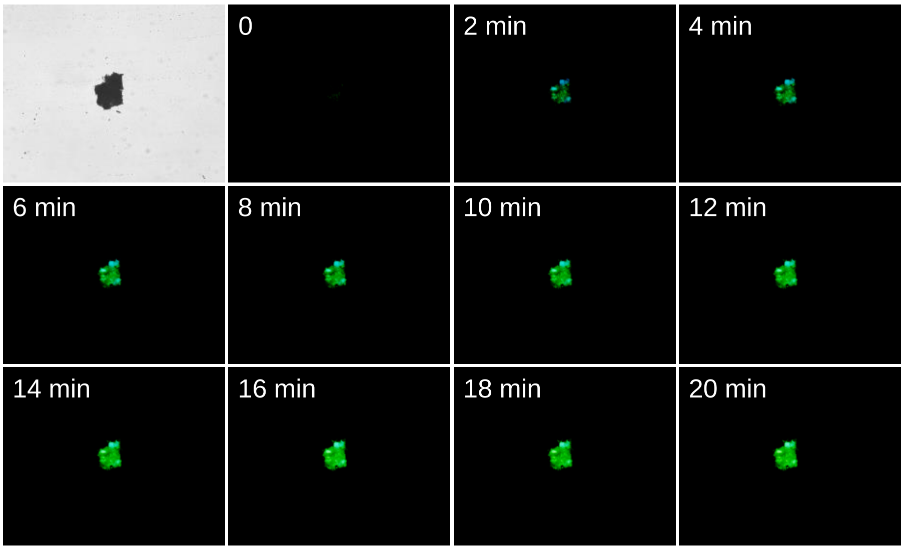

Fig. S14 Bright field and fluorescence images of **3b** in the solid state taken under white light and UV irradiation (365 nm).

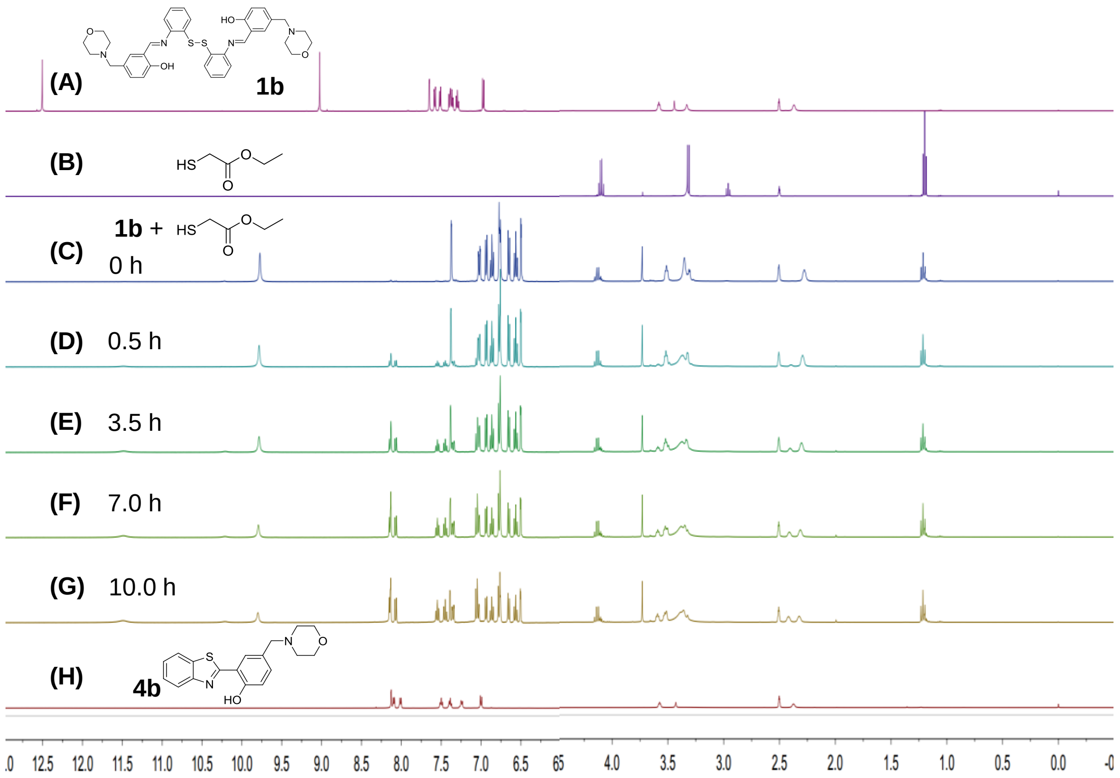

Fig. S15 The ^{1}H NMR spectra for in situ monitoring of the reaction between **1b** and ethyl mercaptoacetate under UV irradiation (365 nm).

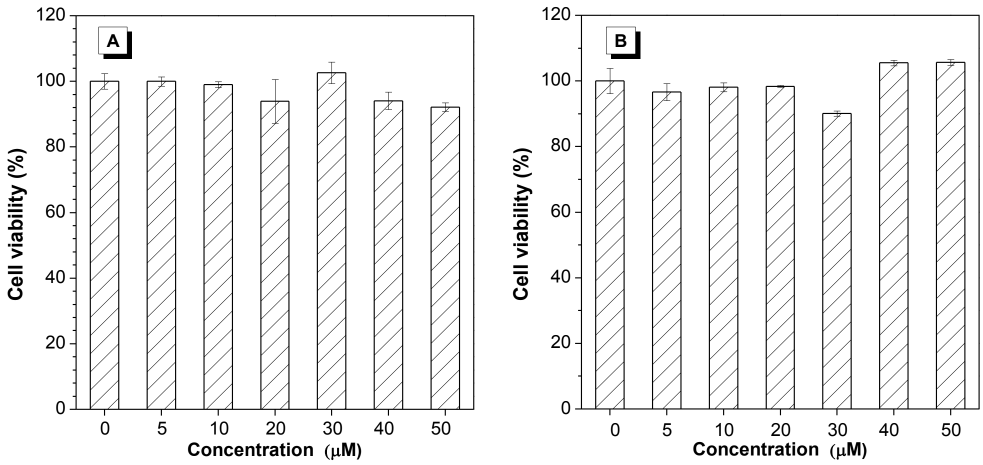

Fig. S16 The cell viabilities of (A) HeLa cells and (B) MCF-7 cells treated with ethyl mercaptoacetate at different concentrations (0, 5, 10, 20, 30, 40, and 50 μM).

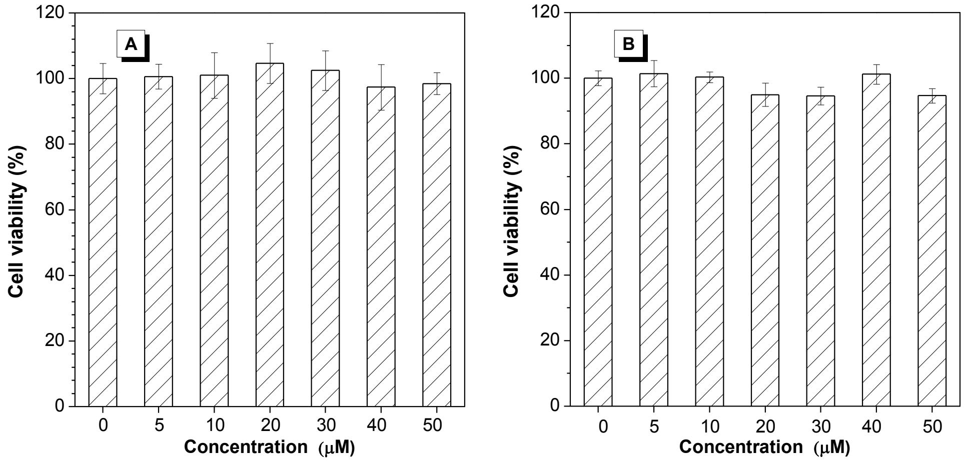

Fig. S17 The cell viabilities of (A) HeLa cells and (B) MCF-7 cells treated with 2,2'-disulfanediyldiacetate at different concentrations (0, 5, 10, 20, 30, 40, and 50 μM).

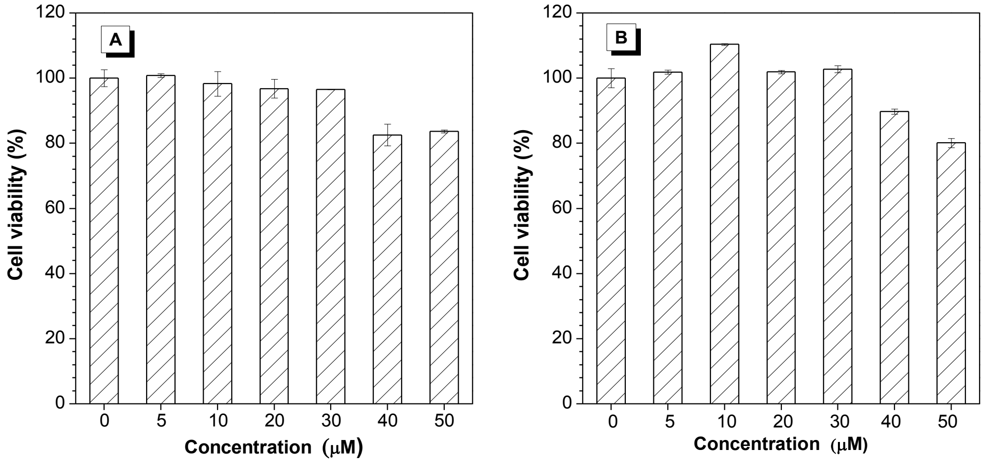

Fig. S18 The cell viabilities of (A) HeLa cells and (B) MCF-7 cells treated with the in situ generated **3a** at different concentrations (0, 5, 10, 20, 30, 40, and 50 μM).

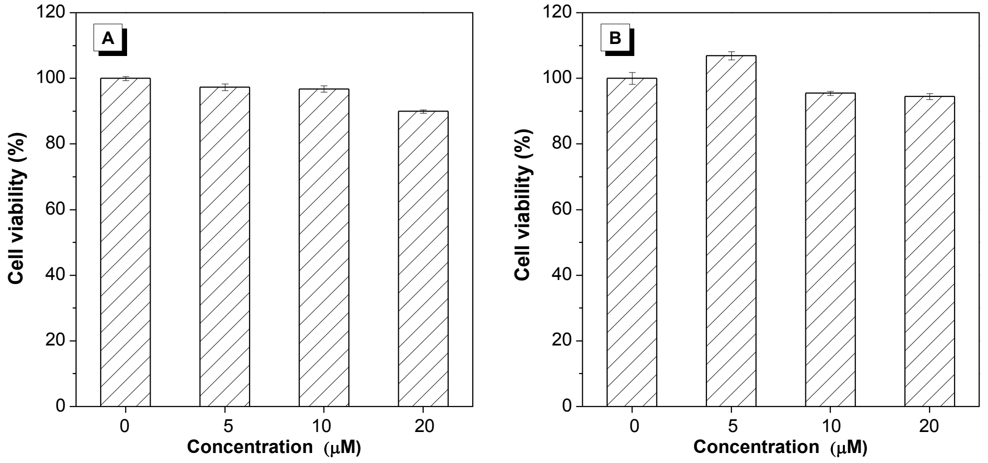

Fig. S19 The cell viabilities of (A) HeLa cells and (B) MCF-7 cells treated with the in situ generated **3b** at different concentrations (0, 5, 10, and 20 μM).

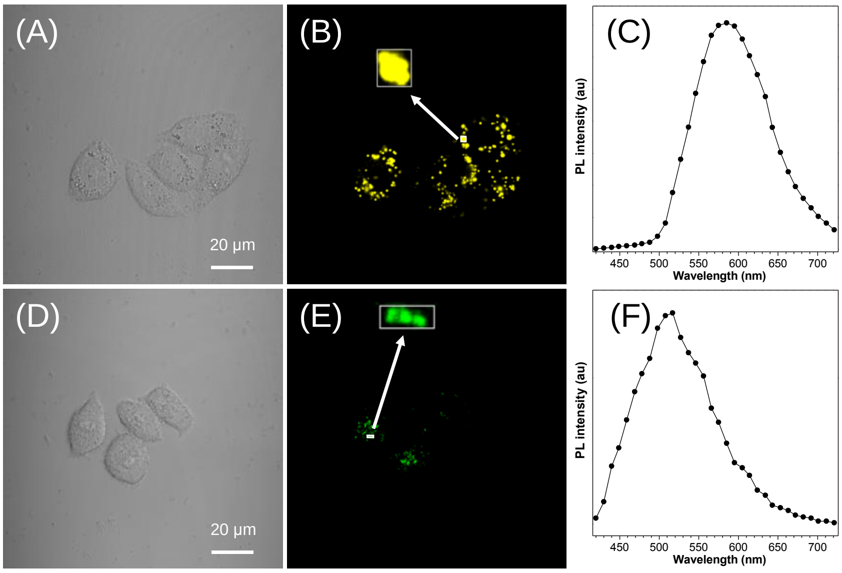

Fig. S20 The bright field and fluorescence images of HeLa cells treated with the in situ generated **3a** (A, B) and **3b** (D, E) after photoactivation by light irradiation at 405 nm; (C) The PL spectra of **3a** in HeLa cells after photoactivation; (F) The PL spectra of **3b** in HeLa cells after photoactivation.

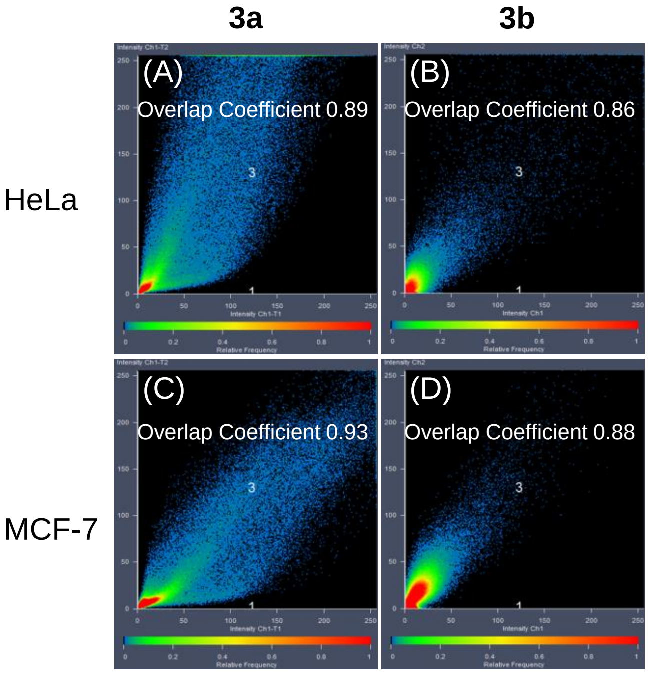

Fig. S21 The intensity correlation plots of **3a** (Y-axis) after photoactivation and BODIPY493/503 Green (X-axis) in (A) HeLa cells (C) and MCF-7 cells. The intensity correlation plots of **3b** (Y-axis) after photoactivation and LysoTracker Red (X-axis) in (B) HeLa cells and (D) MCF-7 cells.

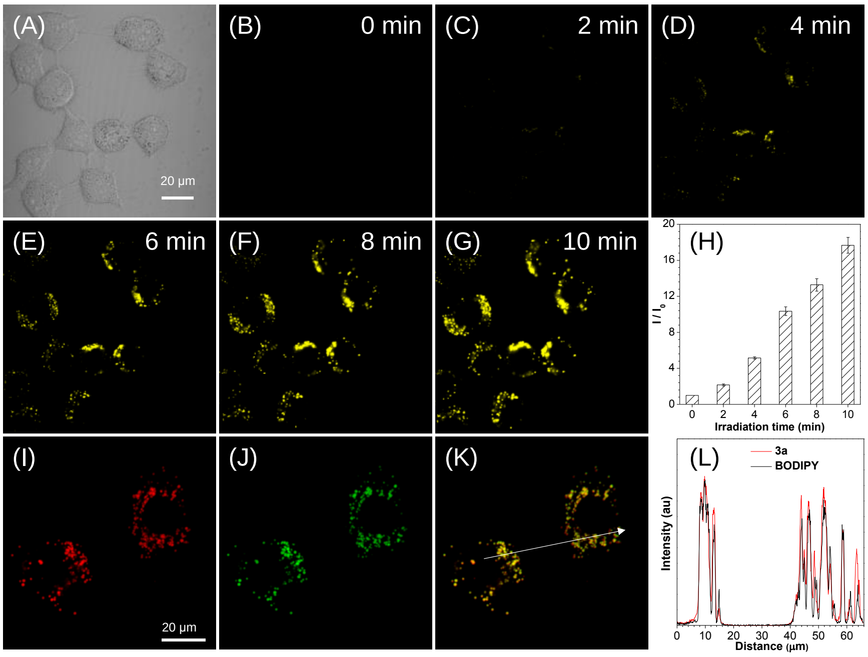

Fig. S22 (A-G) Bright field and fluorescence images of live MCF-7 cells taken under white light and prolonged irradiation at 405 nm. (H) Plot of fluorescence enhancement (I/I_0) of MCF-7 cells with increasing irradiation time at 405 nm. (I-K) Fluorescence images of MCF-7 cells stained with **3a** after photoactivation (red), BODIPY493/503 (green), and the merged image. (L) The intensity profile of ROI lines. [**3a**] = 50 μM.

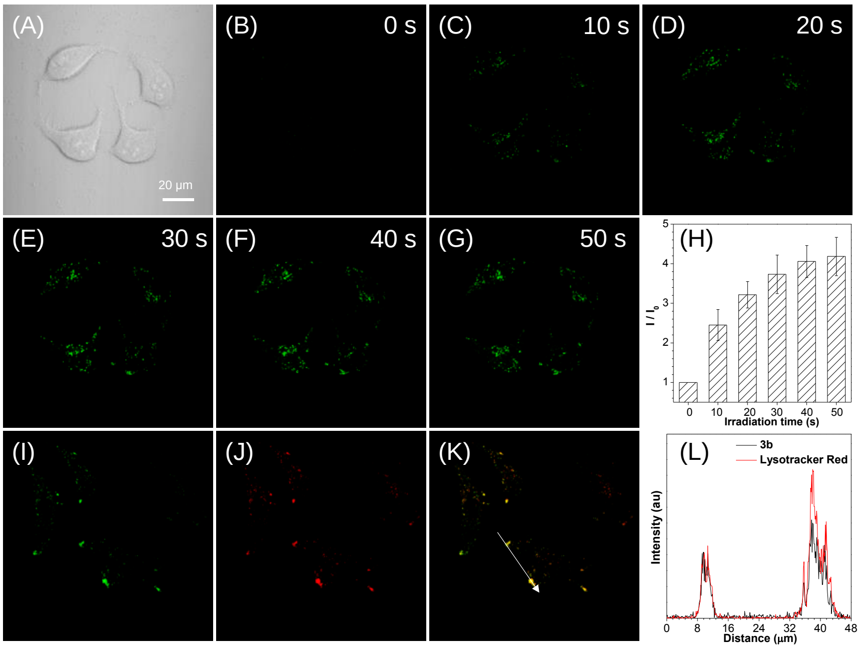

Fig. S23 (A-G) Bright field and fluorescence images of live MCF-7 cells taken under white light and prolonged irradiation at 405 nm. (H) Plot of fluorescence enhancement (I/I_0) of MCF-7 cells with increasing irradiation time at 405 nm. (I-K) Fluorescence images of MCF-7 cells stained with **3b** after photoactivation (green), LysoTracker Red (red), and the merged image. (L) The intensity profile of ROI lines. [**3b**] = 20 μM.

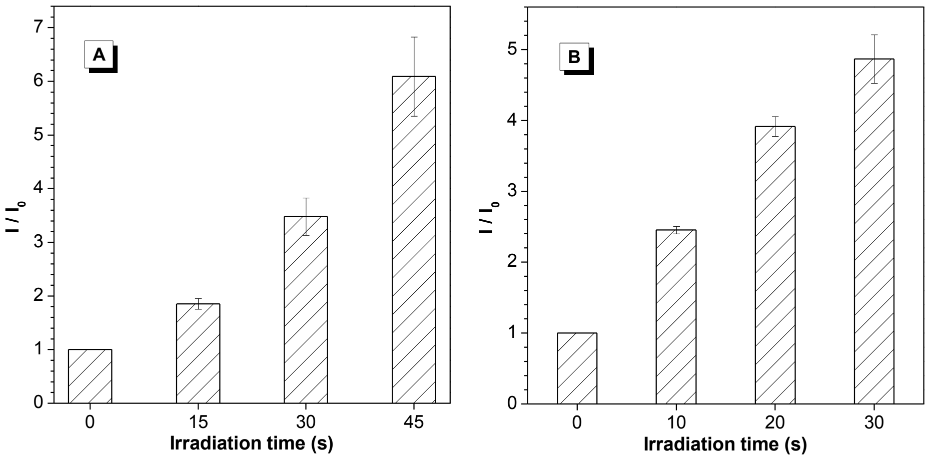

Fig. S24 Plots of fluorescence enhancement (I/I_0) of HeLa cells treated with in situ generated (A) **3a** and (B) **3b** under two-photon irradiation for prolonged time. [**3a**] = 50 μM; [**3b**] = 20 μM.

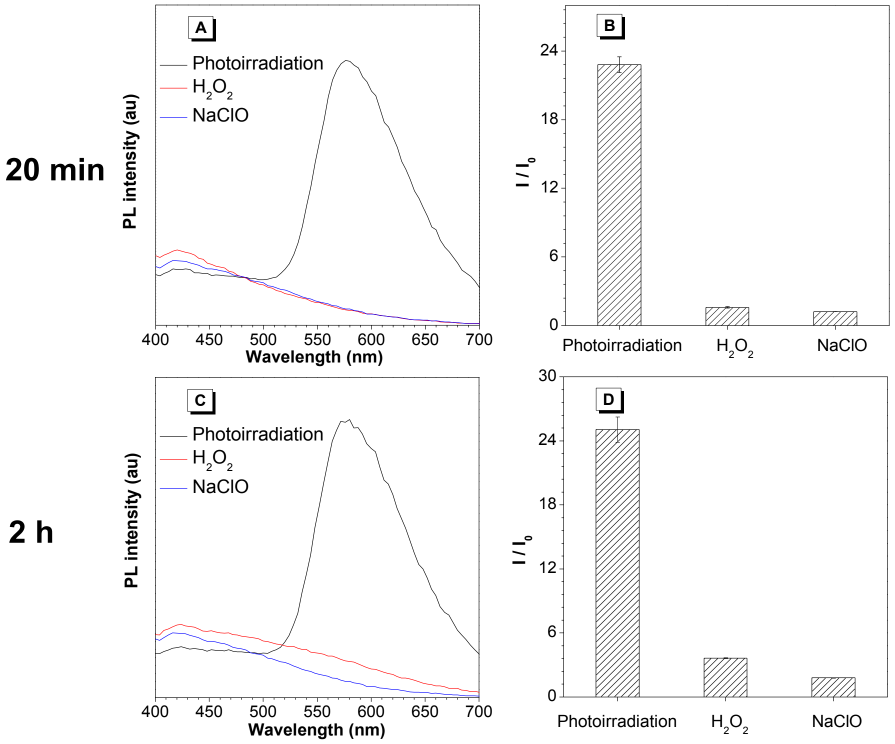

Fig. S25 (A, C) The PL spectra and (B, D) light-up ratios at 570 nm for the in situ generated **3a** in aqueous solution and treated with UV irradiation (365 nm), H_2O_2, and NaClO for 20 min and 2 h, respectively. [H_2O_2] = [NaClO] = 100 μM.

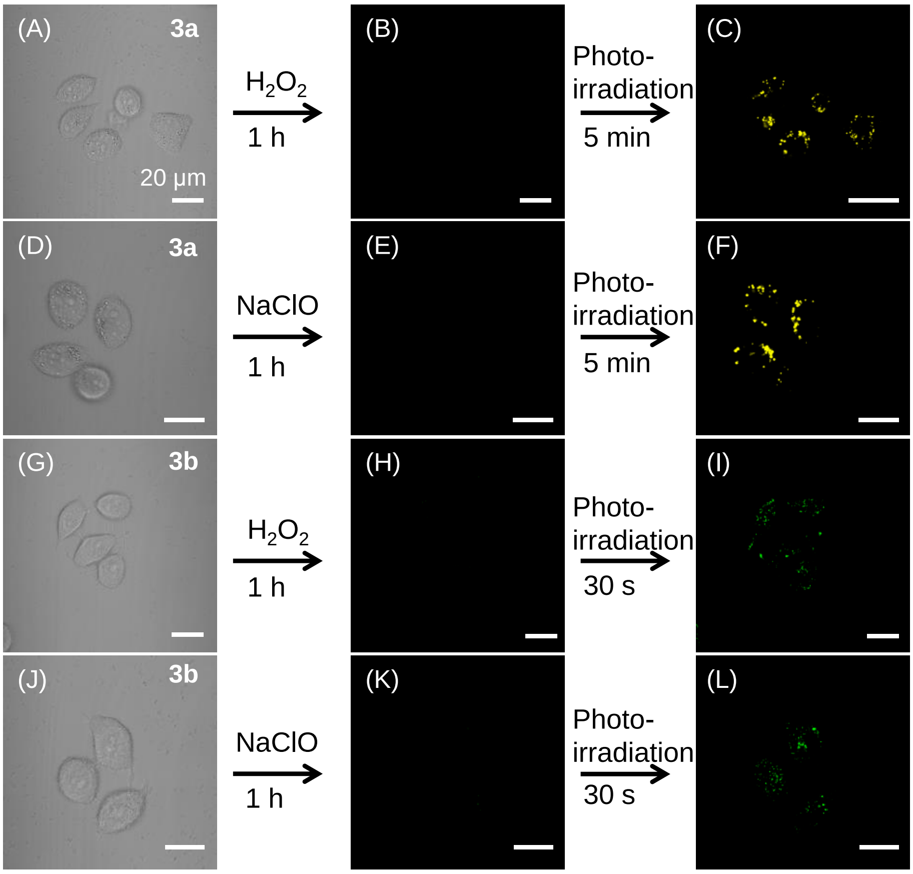

Fig. S26 Bright field and fluorescence images of HeLa cells stained with the in situ generated **3a** and **3b**. Bright field images (A, D, G, and J); fluorescence images after incubation with H_2O_2 or NaClO for 20 min (B, E, H and K); fluorescence images after photo-irradiation (405 nm, 10% laser power) for 5 min (C and F); fluorescence images after photo-irradiation (405 nm, 5% laser power) for 30 s (I and L). [**3a**] = 50 μM; [**3b**] = 20 μM; [H_2O_2] = [NaClO] = 100 μM.

References

(1) S. T. Chew, K. M. Lo, S. K. Lee, M. P. Heng, W. Y. Teoh, K. S. Sim and K. W. Tan, *Eur. J. Med. Chem.*, 2014, **76**, 397-407.
(2) X. Wei, J. Li, B. Zhou and S. Qin, *Transit. Metal Chem.*, 2004, **29**, 457-462.
(3) K. Yamamoto, M. Fujita, K. Tabashi, Y. Kawashima, E. Kato, M. Oya and T. Iso, J. Iwao, *J. Med. Chem.*, 1988, **31**, 919-930.
(4) P. Raj, A. Singh, K. Kaur, T. Aree, A. Singh and N. Singh, *Inorg. Chem.*, 2016, **55**, 4874-4883.
